# DNA microbeads for spatio-temporally controlled morphogen release within organoids

**DOI:** 10.1038/s41565-024-01779-y

**Published:** 2024-09-09

**Authors:** Cassian Afting, Tobias Walther, Oliver M. Drozdowski, Christina Schlagheck, Ulrich S. Schwarz, Joachim Wittbrodt, Kerstin Göpfrich

**Affiliations:** 1 Centre for Organismal Studies Heidelberg (COS), Heidelberg University, Heidelberg, Germany; 2 Heidelberg International Biosciences Graduate School HBIGS, Heidelberg, Germany; 3 HeiKa Graduate School on “Functional Materials”, Heidelberg, Germany; 4 Center for Molecular Biology of Heidelberg University (ZMBH), Heidelberg University, Heidelberg, Germany; 5 Biophysical Engineering Group, Max Planck Institute for Medical Research, Heidelberg, Germany; 6 BioQuant Center, Heidelberg University, Heidelberg, Germany; 7 Institute for Theoretical Physics, Heidelberg University, Heidelberg, Germany; 8 Max Planck School Matter to Life, Heidelberg, Germany

**Keywords:** Biomaterials, DNA nanotechnology

## Abstract

Organoids are transformative in vitro model systems that mimic features of the corresponding tissue in vivo. However, across tissue types and species, organoids still often fail to reach full maturity and function because biochemical cues cannot be provided from within the organoid to guide their development. Here we introduce nanoengineered DNA microbeads with tissue mimetic tunable stiffness for implementing spatio-temporally controlled morphogen gradients inside of organoids at any point in their development. Using medaka retinal organoids and early embryos, we show that DNA microbeads can be integrated into embryos and organoids by microinjection and erased in a non-invasive manner with light. Coupling a recombinant surrogate Wnt to the DNA microbeads, we demonstrate the spatio-temporally controlled morphogen release from the microinjection site, which leads to morphogen gradients resulting in the formation of retinal pigmented epithelium while maintaining neuroretinal cell types. Thus, we bioengineered retinal organoids to more closely mirror the cell type diversity of in vivo retinae. Owing to the facile, one-pot fabrication process, the DNA microbead technology can be adapted to other organoid systems for improved tissue mimicry.

## Abstract (web summary)

Creating precise morphogen gradients for tissue engineering is challenging. Here the authors present mechanically tunable DNA hydrogel-based microbeads for light-controlled morphogen release in retinal organoids for better tissue mimicry.

## Main

Organoids have become a widely used tool in basic research, human disease modelling and personalized medicine, and have been established for a variety of organs^1^. Retinal organoids (RO) specifically have been assembled and studied from mice, humans and fish. Among them, medaka (*Oryzias latipes*) fish RO develop by far the fastest and can be derived from easily generated transgenic reporter lines^2–4^, making them particularly well suited for the development of new tissue engineering technologies. While organoids, including RO, share many of their properties with their in vivo counterparts, end-point morphology, cell type diversity and functionality have proven difficult to replicate. The lack of spatial organization of morphogen gradients is one of the vital factors limiting the organoid’s full emulation of the respective organ and keeping them from being a more physiologically relevant model system^1^. Using engineered materials for spatio-temporal delivery of bioactive cues to ultimately guide organoid development could be a promising avenue to address these limitations^5,6^.

Thus far, morphogen gradients have mainly been implemented in stem cell culture by microfluidic devices^7–10^, patterning of hydrogels with biochemical cues^11–14^ and integration of transgenic cellular signalling centres at an organoid’s pole^15^. Yet, these approaches can only provide unidirectional slopes of morphogen gradients from the outside to the inside of a respective organoid, constantly exposing the outer cell layers of an organoid to higher concentrations of morphogens than the inner cell layers. To create spatially discrete, organoid-internal morphogen sources and thus reversed gradients, the utility of micro-/nanoparticles in co-aggregation during early spheroid assembly has been explored previously^16–19^. Utilizing stem cell aggregate merging techniques, broad spatial control over microparticle-mediated morphogen release in merged aggregates has been achieved^19^. Nevertheless, this technique gives the user neither direct and precise spatial nor temporal control; it is optimized for early organoid assembly and has only limited, if any, applicability in mid- to late-stage organoid culture. As such, better control over the onset of morphogen gradients and new and broadly applicable techniques for morphogen delivery are needed.

DNA hydrogel materials have gained popularity owing to their simple programmability via sequence specificity^20,21^. In this way, versatile DNA-based materials with controllable stiffness^22–24^ and chemical modification, for example, by click chemistry^25^ with pH^25,26^ or light responsivity^27–29^ have been created, including DNA droplets that form by liquid–liquid phase separation^30–34^. Such droplets have been used as tools for the uptake and delivery of molecular cargo^30,35,36^ even in living systems^25,31,37^. However, apart from the formation of DNA-based hydrogels as an extracellular matrix mimic^38^, the potential of DNA hydrogels as a tool for the engineering of organoids remains largely unexplored.

In this Article, we present DNA microbeads as a modifiable DNA hydrogel material that can be integrated via microinjection as a spatially discrete and temporally controllable source of morphogen gradients inside of an organoid at any point in its life cycle. Microinjected DNA microbeads do not influence normal organoid development and are non-invasively erasable after tissue integration by light-triggered breakdown. By creating RO internal gradients of a Wnt agonist, we engineer RO more closely mirroring the cell type diversity of the in vivo retina, exemplifying how the presented tool can increase the complexity and phenotypic accuracy of organoid culture.

## Customizable material properties of DNA microbeads

To establish a generalizable tool capable of providing chemical cues from within the organoid, we set out to engineer DNA microbeads, which fulfil several key requirements: (i) scalability (the DNA microbead production should be simple and scalable, without the use of specialized equipment or expert knowledge, such that it can be performed in any laboratory), (ii) tunable mechanics (the mechanical properties of the DNA microbeads must be tunable to mimic a diverse range of cell stiffnesses or to provide mechanical cues), (iii) microinjection compatible (the DNA microbeads must be highly resistant to shear stress to allow microinjection into organoids), (iv) biocompatibility (the microbeads should be stable in the organoids’ interior and degradable on demand once they served their purpose to avoid undesired influences on organoid development) and (v) chemically modifiable (it must be possible to attach multiple chemical cues onto the microbeads and release them on demand spatio-temporally controlled within the organoid’s interior).

**Fig. 1:**
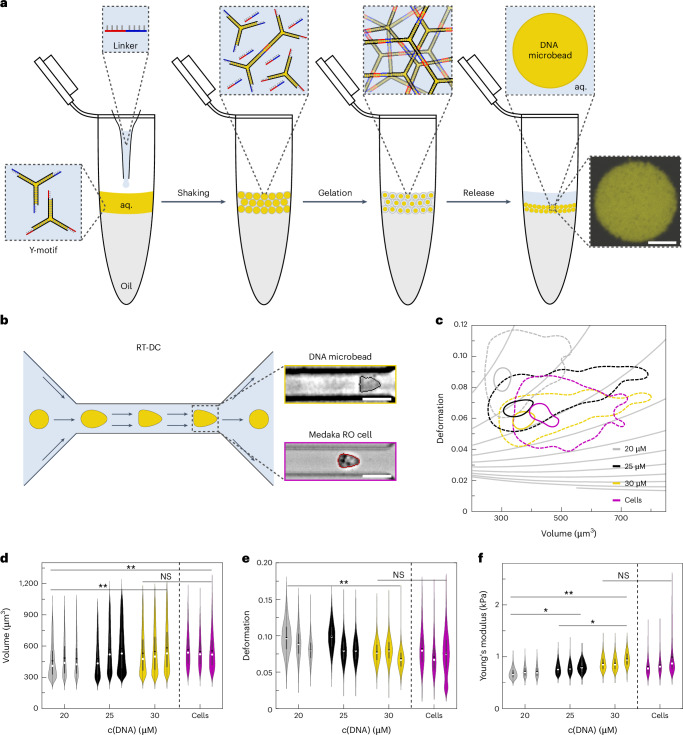
DNA microbead production and stiffness adaptation to RO cells. **a**, Scheme of the DNA microbead production (aq., aqueous solution). Right: confocal microscopy image of a DNA microbead (*λ*_ex_ = 561 nm, Cy3-labelled DNA). Scale bar, 10 µm. **b**, Scheme of RT-DC for high-throughput stiffness characterization. DNA microbeads and RO cells are flushed through a microfluidic channel (width 20 µm) and deform under shear stress. Brightfield images showing a deformed DNA microbead and RO cell, respectively. Scale bars, 20 µm. **c**, Plot showing the deformation of different populations of DNA microbeads and RO cells over the corresponding volume using contour plots showing the 50th percentile (dashed line) and 95th percentile (solid line) of each measurement. **d**, Volume of the measured DNA microbeads and RO cells. Statistically significant differences were detected for 20 µM and 30 µM DNA microbeads (**P value 0.002), and 20 µM DNA microbeads and RO cells (**P value 0.001). No statistically significant differences were detected for 30 µM DNA microbeads (*n*_20µM_ = 32028, *n*_25µM_ = 41137, *n*_30µM_ = 38254, individual particles measured) and RO cells (*n*_Cells_ = 25853). **e**, Deformation of the DNA microbeads and RO cells. Statistically significant differences were detected for 20 µM and 30 µM DNA microbeads (**P value 0.008). No statistically significant differences were detected for 30 µM DNA microbeads and RO cells. **f**, Apparent Young’s moduli of the DNA microbeads and RO cells. Statistically significant differences were detected for 20 µM and 25 µM DNA microbeads (*P value 0.01), 20 µM and 30 µM DNA microbeads (**P value 0.001), 25 µM and 30 µM DNA microbeads (*P value 0.02) and 20 µM DNA microbeads and RO cells (*P value 0.035). No statistically significant differences were detected for 30 µM DNA microbeads and RO cells. Statistical significance was assessed using a linear mixed model without adjustments (R-lme4) as integrated in Shape-Out (version 2.10.0). Statistical significance was assessed via ANOVA test. For each dataset (**d**–**f**), the data distribution is shown as a violin plot, depicting the median (white circle) and mean value (black line). Box plots depict the 25–75% percentiles with a whisker length of 1.5 IQR, interquartile range.

Hence, we first designed DNA microbeads and experimentally confirmed that they fulfil Requirements (i–v). We followed a DNA design consisting of three single strands, which bind to form branched, double-stranded DNA nanostructures with three arms termed Y-motif^39^ (Fig. 1a). These DNA nanostructures can form DNA hydrogels, when interlinked via short sticky-end overhangs at each end of the Y-motif arms^29,40^. We used two Y-motifs with orthogonal sticky-end overhangs. Upon addition of a single-stranded piece of DNA complementary to both sets of sticky-end sequences (DNA linker), these Y-motifs form a hydrogel network^29,40^. We realized that encapsulating the DNA strands into water-in-oil droplets allows for the droplet-templated formation of micrometre-sized DNA hydrogel beads (Fig. 1a). In brief, the aqueous solution containing the two orthogonal Y-motifs and the DNA linker is layered on top of an oil–surfactant solution in a reaction tube. A droplet emulsion is created by manual shaking of the reaction tube. The DNA condenses into microbeads by self-assembly. In the final step, the emulsion is broken up and the ready-to-use DNA microbeads are released into an aqueous phase^41^. This facile formation stably produces large quantities of DNA microbeads in a one-pot reaction without specialized equipment within minutes of manual labour, making them an easy tool to implement in any laboratory. A single production produces enough material for several hundred organoid microinjections. We confirm that the DNA microbeads are stable after microcentrifugation and pelleting, which allows for facile buffer exchange. The microbeads form a gel-like network as confirmed by fluorescence recovery after photobleaching experiments and can be stored in the fridge for at least 1 year (Supplementary Fig. 1). We thus validated that our method for forming DNA microbeads fulfils all aspects of Requirement (i).

Tools for the engineering of living systems need to be tailored to accommodate stiffness parameters to mitigate unwanted effects or to provide mechanical cues on demand. As such, we set out to test whether the DNA microbeads can be tuned to match the stiffness of organoid cells. We utilized real-time deformability cytometry (RT-DC) as a high-throughput microfluidic method to analyse the apparent Young’s modulus of RO cells and DNA microbeads, as so far only the stiffness of bulk DNA hydrogels had been characterized^22–24^. This way, we investigated if we can fine-tune the properties of the DNA microbeads to match the organoid cells^42,43^ (Fig. 1b). We assumed that the stiffness of the DNA microbeads can be tuned by varying the concentration of the Y-motifs (20 µM, 25 µM and 30 µM). RT-DC experiments revealed an increase in overall DNA microbead volume and a decrease in deformation with higher DNA-Y-motif concentration (Fig. 1c–e). While significant differences in volume were found between several DNA-Y-motif concentrations, average overall volumes of RO cells and 30 µM DNA microbeads were almost identical (Fig. 1d and Supplementary Table 1). We obtained a similar result for the deformation of the different DNA microbeads and RO cells, as the 30 µM DNA microbeads and the RO cells again showed almost identical average values (Fig. 1e and Supplementary Table 2). Finally, analysing the apparent Young’s moduli of the different DNA microbeads and cell samples, significant differences were detected between all three of the tested DNA-Y-motif concentrations. No significant difference in apparent stiffness was detected between the 30 µM DNA microbeads and the RO cells, both showing almost identical average values (Fig. 1f and Supplementary Table 3). We confirmed the stiffness values of the DNA microbeads obtained from RT-DC with microindentation experiments (Supplementary Fig. 2). We thus confirmed that the DNA microbeads exhibit mechanical tunability fulfilling Requirement (ii). Owing to the excellent match of the mechanical properties with the organoid cells, we selected the 30 µM DNA microbeads to be used in all further experiments.

## DNA microbead delivery into retinae and RO

**Fig. 2:**
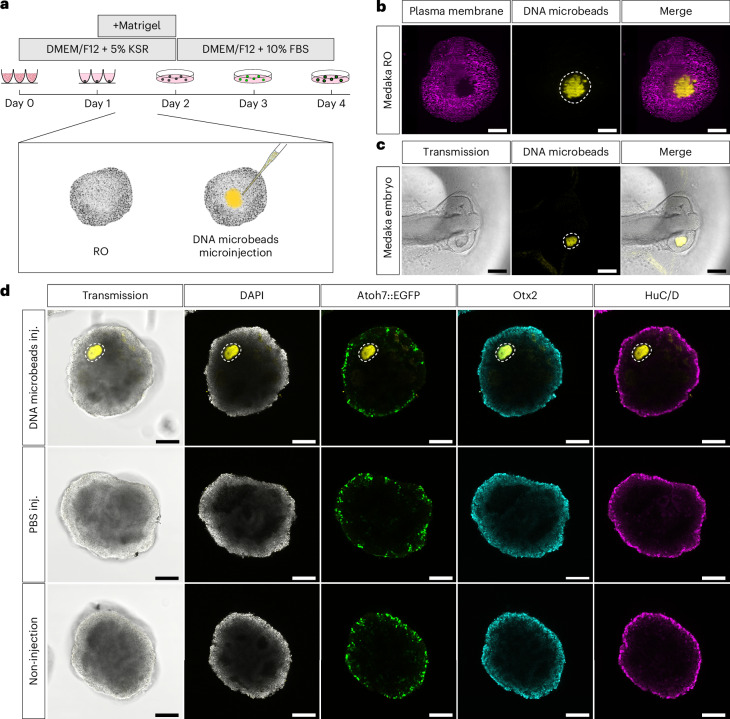
DNA microbead delivery and integration into the developing in vitro and in vivo medaka retina. **a**, Schematic illustration of medaka RO generation and time point of DNA microbead microinjection. **b**, Light sheet fluorescence microscopic image of a plasma membrane-stained day 3 RO post microinjection with DNA microbeads. **c**, Transmission image of an alive stage 20 medaka embryo 2 h after DNA microbead microinjection into its developing retina. **d**, Representative confocal images of whole-mount antibody-stained day 4 RO (DAPI (nuclei), Atoh7::EGFP (retinal ganglion cells), Otx2 (bipolar cells and photoreceptors) and HuC/D (amacrine and retinal ganglion cells)) after microinjection with DNA microbeads (DNA microbeads inj.; *λ*_ex_ = 561 nm, Cy3-labelled DNA), PBS (PBS inj.) or being left uninjected (non-injection). Dashed white lines outline the DNA microbeads’ positions. Representative images from *n* = 25 organoids across 3 independent experiments. Scale bars, 100 µm.

Having demonstrated that DNA microbeads can be produced in a scalable manner and have suitable mechanical properties, we test whether they are sufficiently stable for microinjection in vivo and in vitro (Requirement (iii)). We thus turned to the fast-developing vertebrate model, medaka fish. For in vitro and in vivo DNA microbead integration, we developed experimental pipelines for microparticle microinjection into RO and embryos. Similar as described previously^3^, we generated RO with a live transgenic reporter labelling retinal ganglion cells and used these as a proxy for the overall formation of neuroretina in the organoids. RO were microinjected with DNA microbeads at late day 1, shortly after Matrigel-induced onset of neuroepithelium formation (Fig. 2a). Light sheet microscopy showed that the DNA microbeads integrated seamlessly into the organoid’s tissue environment (Fig. 2b). Note that the DNA microbeads withstand the strong shear forces during microinjection without disintegration and remain within the organoid system even under changing culture media and conditions (Supplementary Fig. 3; Requirement (iv)). Likewise, microinjected DNA microbeads were integrated into the corresponding developmental stage of the in vivo embryonic medaka retina (s20 (ref. ^44^); Fig. 2c). Culturing microinjected organoids until differentiation onset at day 4 showed that the DNA microbeads were stable within the organoids (Fig. 2d), while they were naturally broken down within the developing retina of the medaka embryo over the course of 6–9 h (Supplementary Fig. 4a). Extracellular DNase activity during early medaka development is likely the reason for this comparably fast natural degradation. This can be considered an asset of the presented technology as it allows for a defined cargo release and DNA microbead removal without the necessity for user intervention. The DNA microbead microinjection thus affected neither the survival nor the gross morphological development of the embryos to hatchling stage compared with non-injected control embryos (s40 (ref. ^44^); Supplementary Fig. 4). For RO, we whole-mount antibody-stained DNA microbead microinjected RO at day 4 with common molecular markers for differentiated retinal cell type identities and imaged them via confocal microscopy. Differentiated retinal cell type composition and patterning did not differ from the respective phosphate-buffered saline (PBS)-microinjected and non-injected controls (Fig. 2d). Thus, neither the presence and integration of DNA microbeads within nor the microinjection into RO seemed to affect their normal development according to the expression and distribution of common molecular markers for differentiated retinal cell types (Requirement (iv)).

## Light-triggered removal of DNA microbeads from organoids

**Fig. 3:**
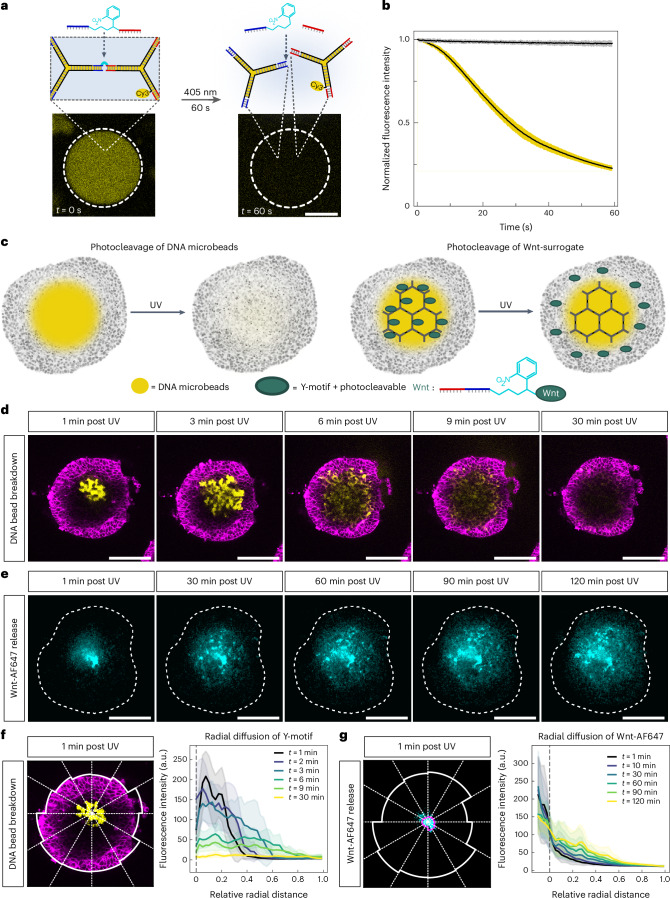
DNA microbeads can be removed from RO non-invasively using light while also allowing local release of Wnt-surrogate in a gradient from the inside to the outside. **a**, Schematic illustration of the DNA microbead design with an internal PC group in the DNA linker sequence. Representative confocal images (*λ*_ex_ = 561 nm, Cy3-labelled DNA) of a PC-modified DNA microbead before and after 60 s illumination with a 405 nm confocal laser (0.5 mW power). The white dashed circle indicates the illuminated area. Scale bar, 20 µm. **b**, Normalized fluorescence signal (mean ± standard deviation, 3 independent replicates analysing 5 DNA microbeads each) plotted over the exposure time of 60 s both for non-PC-modified DNA microbeads (grey line) and PC-modified DNA microbeads (yellow line). **c**, Left: schematic illustration of PC-modified DNA microbead breakdown within RO. Right: schematic illustration of the DNA microbead design with photoinducible Wnt-surrogate release from intact DNA microbeads. **d**, Representative time-lapse confocal imaging of Cy3-Y-motif fluorescent signal (yellow) after microinjection and subsequent breakdown of PC-modified DNA microbeads in live small RO. Small RO are counterstained with live plasma membrane stain (magenta). For full time-lapse, see Supplementary Video 1. **e**, Representative time-lapse confocal imaging of Wnt-surrogate tagged with Alexa Fluor 647 (Wnt-AF647) after release from DNA microbeads in live small RO. Images show a maximum intensity *z*-projection of 10 slices spaced 3 µm. *z*-Projections were despeckled for noise reduction. Dotted white lines indicate the small RO shape. For full time-lapse, see Supplementary Video 2. Scale bars, 100 µm. **f**, Quantification of the radial diffusion of Cy3-Y-motif fluorescent signal after DNA microbead breakdown within live small RO (*n* = 1 organoid was considered with 12 slices; lines represent the average intensity with error bands showing point-wise standard deviation). **g**, Quantification of the radial diffusion of Wnt-AF647 after release within live small RO visualizing the formation of an inside-out gradient (*n* = 1 organoid was considered with 12 slices; lines represent the average intensity with error bands showing point-wise standard deviation).

Having confirmed that DNA microbeads are stable inside medaka RO and do not hinder their development, we next incorporated functionality into the DNA microbeads as a means of controlling their behaviour inside the organoids to meet Requirement (iv). Adding a photocleavable (PC) moiety to the centre of the DNA linker^29^ allowed for the near-instantaneous breakdown of the DNA microbeads upon irradiation with 405 nm light with spatio-temporal control (PC-modified DNA microbeads; Fig. 3a,b and Supplementary Fig. 5). We confirmed that the light-triggered breakdown of PC-modified DNA microbeads is possible not only in bulk solution but also in microinjected RO (Fig. 3c). Following ultraviolet (UV) irradiation, the fluorescent signal of the DNA microbeads can be observed to disappear from inside of the RO within approximately 25–30 min (Supplementary Fig. 6). Therefore, using the PC modification on DNA microbeads allows for their non-invasive removal after tissue integration with full user control. Whole-mount antibody staining and confocal microscopy showed no difference in retinal cell type composition and patterning of day 4 RO after PC DNA microbead breakdown compared with controls with and without UV light treatment (Supplementary Fig. 7). Accordingly, neither the UV light regime nor the release of free DNA motifs negatively affected normal medaka RO development.

## Controlled morphogen gradients within organoids

Next, we demonstrated the utility of the DNA microbead system to form gradients of chemical cues within organoids following targeted release of a morphogen (Requirement (v)). Particularly, we aimed at releasing a Wnt agonist as an exemplary morphogen from the DNA microbeads within the RO. Wnt agonists are frequently used in RO cell culture as they are known to induce retinal pigmented epithelium (RPE) formation, which otherwise occurs rarely and insufficiently. Beyond RO, Wnt constitutes an essential player in developing organs and organoids across types and species and is widely used in several organoid cell cultures^45^.

Here we used an extracellularly binding, next-generation surrogate Wnt^46^ (Wnt-surrogate), which we covalently attached via a PC group to the DNA linker using bio-orthogonal DBCO-azide click chemistry^47^ (Supplementary Fig. 8). This allowed for the incorporation of Wnt-surrogate into the DNA microbeads (Supplementary Figs. 9–11). Owing to its small size, 5-FAM-modified DNA linkers readily incorporate into the DNA network upon mixing (Supplementary Fig. 10a,b), while larger cargo such as Wnt-surrogate gets incorporated following overnight incubation (Supplementary Fig. 11a,b). Owing to their smaller size, 5-FAM molecules diffuse faster out of the DNA microbeads than Wnt-surrogate following photocleavage (Supplementary Figs. 10c and 11c).

Combining Wnt-surrogate DNA microbead modification with microinjection into live RO, Wnt-surrogate was released from the DNA microbeads upon irradiation with UV light without DNA microbead breakdown (Wnt-DNA microbeads; Fig. 3c). To investigate the spatio-temporal dynamics of Wnt-surrogate released in RO, we conjugated a small, highly photostable fluorescent tag (Alexa Fluor 647; AF647) onto it. Since the size of standard-sized RO was found to be too big to allow for proper confocal laser penetration with live-imaging-compatible laser intensities, we reduced the size of the RO to reliably assess the spatio-temporal dynamics. Reducing seeding cell numbers creates smaller RO (2/3 of the diameter of standard-sized ones at day 1) while maintaining the overall morphology, retinal cell type diversity and patterning observed in standard-sized RO (ref. ^3^) (Supplementary Fig. 12).

We performed confocal time-lapse imaging of Wnt-surrogate-AF647 (Wnt-AF647) after release from the DNA microbeads and compared it with confocal time-lapse imaging of the DNA microbeads’ fluorescence signal (Cy3-tagged Y-motifs) after DNA microbead breakdown in small RO. The Wnt-surrogate diffusion distributed markedly slower and in a locally punctuated fashion compared with the fast and globally more uniform distribution of the Cy3-tagged Y-motif (Fig. 3d,e and Supplementary Videos 1 and 2). As expected, Wnt-surrogate distributed in a gradient from the inside towards the outside of the small RO (Fig. 3f,g). The locality of the Wnt-AF647 diffusion can be expected to be aggravated in the standard-sized RO. Wnt diffusion in the extracellular space of tissues is known to be influenced by a multitude of factors such as constant binding and unbinding to heparan sulfate proteoglycans^48,49^ and assembly and dissociation of protein complexes^50^. This and Wnt-surrogate binding to its target receptors on cellular plasma membranes explain the differences observed to the diffusion dynamics and pattern of the Cy3-tagged Y-motif, which do not interact specifically with the organoid. If the DNA microbead deposit was microinjected close to the edge of the small RO, Wnt-AF647 diffusion was restricted to one of its sides, emphasizing the spatial control ability of the presented technology (Supplementary Fig. 13). To confirm that the formation of the different gradients observed experimentally can be explained by differences in diffusion, interaction with the organoid tissue and the known conditions of release from the DNA microbeads, we simulated a corresponding three-dimensional diffusion-degradation model, similar to earlier theory work on morphogen gradients and interferon signalling^51,52^. We identified parameter values that robustly reproduced the experimentally observed spatio-temporal gradient dynamics (Supplementary Note 1 and Supplementary Fig. 14). Our theory showed that the two cases of Wnt-surrogate and DNA-Y-motif gradients correspond to the two different regimes of release-limited and diffusion-limited spreading, respectively (Supplementary Fig. 15). Together, these results confirm that the DNA microbead technology establishes gradients in a physically controlled manner that can be adapted to desired applications.

## Internal morphogens bioengineer more in vivo-like organoids

Finally, we demonstrated the utility of the DNA microbead system to guide organoid development via the targeted release of a morphogen in a gradient from the organoids inside towards its outside (Requirement (v)). Currently, the on-demand induction of RPE in RO with Wnt agonists supplemented to the culture medium results in the unwanted suppression of neuroretinal tissue^2^ and therefore does not permit the full emulation of the in vivo retinal cell type diversity in RO cell culture across species.

**Fig. 4:**
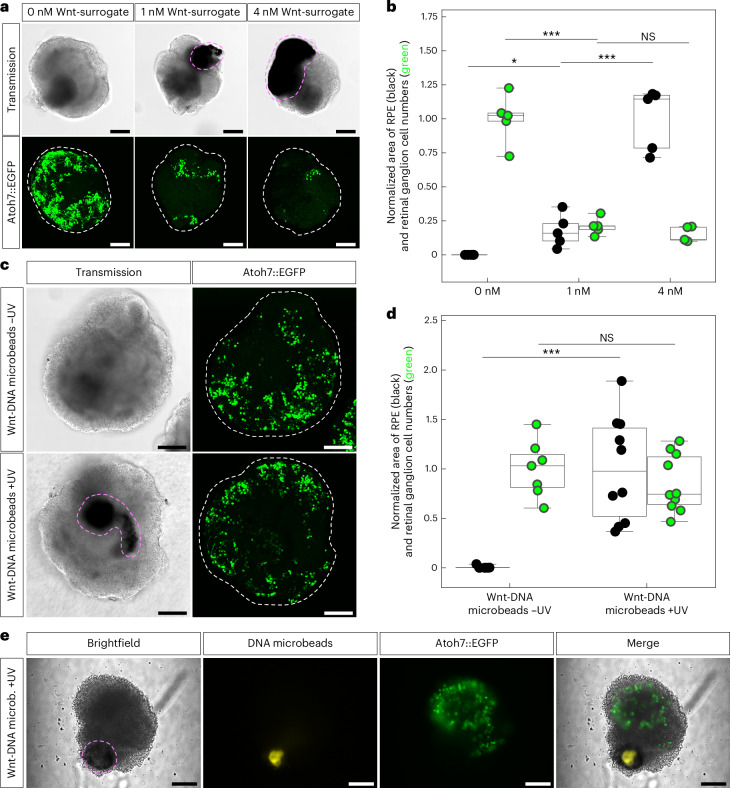
The controlled release of Wnt-surrogate in an organoid internal gradient permits the bioengineering of RO with a more in vivo-like retinal cell type diversity. **a**, Representative confocal transmission and maximum intensity *z*-projection (Atoh7::EGFP; 35 slices at 5 µm distance) images of day 4 RO treated with 0 nM, 1 nM and 4 nM Wnt-surrogate in the culture medium. **b**, Quantification of the area of RPE (black) and retinal ganglion cell numbers (green) from representative transmission and Atoh7::EGFP maximum intensity *z*-projection images obtained as in **a**. Each box plot contains data from *n* = 5 RO. **c**, Representative confocal transmission and maximum intensity *z*-projection (Atoh7::EGFP; 15 slices at 10 µm distance) images of day 4 RO after Wnt-DNA microbead microinjection and Wnt-surrogate release at day 1. White dashed lines outline the shape of the respective RO. **d**, Quantification of the area of RPE (black) and retinal ganglion cell numbers (green) from representative transmission and Atoh7::EGFP maximum intensity *z*-projection images obtained as in **c**. Each box plot contains data from *n* = 7 RO for Wnt-DNA microbeads −UV and *n* = 10 RO for Wnt-DNA microbeads +UV. Boxes indicate 25–75% percentiles and whiskers 10–90% percentiles. The central horizontal line indicates the median. Individual data points shown as dots. Retinal ganglion cell numbers were normalized to the average of all data points of the 0 nM or Wnt-DNA microbeads −UV group, while the area of RPE was normalized to the average of all data points of the 4 nM or Wnt-DNA microbeads +UV group. Two-tailed Student’s *t*-tests were performed with unequal variance (NS, not significant; *P value 0.03; 0 nM versus 1 nM ***P value 0.0003; 1 nM versus 4 nM ***P value 0.0004; Wnt-DNA microbeads +/−UV ***P values 0.0002). **e**, Live epifluorescence microscopy of a day 4 RO after Wnt-DNA microbead microinjection and Wnt-surrogate release at day 1 near the RO’s edge. Note that the RPE induction phenotype was deliberately reduced by changes in culture conditions to more precisely show the spatial relationship of the DNA microbeads and RPE differentiation. Magenta dashed lines indicate RPE. Scale bars, 100 µm.

In accordance with literature on other agonistic Wnt molecules, supplementing increasing concentrations of Wnt-surrogate to the medium at day 1 of RO culture resulted in increasing amounts of RPE in the organoids while at the same time heavily suppressing neuroretinal differentiation as visualized by the occurrence of retinal ganglion cells (Fig. 4a,b). With microinjection of Wnt-DNA microbeads into and subsequent release of the Wnt-surrogate within the RO, we induce RPE formation while not suppressing retinal ganglion cells (Fig. 4c,d and Supplementary Fig. 16). This is best explained by the organoid internal Wnt-surrogate gradient limiting the exposure of the neuroretinal cells residing near the rim of the RO, differing from the standard approach of adding Wnt agonists to the culture medium and thus exposing the outer cells most. Our DNA microbead technology consequently enables the bioengineering of RO with a cell type composition more closely mimicking the in vivo retina. Note that the addition of Wnt-surrogate into the DNA microbeads did not significantly alter their stiffness (Supplementary Fig. 17 and Supplementary Tables 4–6).

We next asked whether RPE differentiation can be induced similarly spatially restricted as the Wnt-AF647 diffusion near the edge of small RO suggested (Supplementary Fig. 13). When the Wnt-DNA microbead deposit was microinjected close to the edge of the RO, RPE was indeed induced only on that side of the RO. In fact, RPE formed directly around the DNA microbeads’ position, confirming the spatial control ability of the presented technology (Fig. 4e, Supplementary Video 3 and Supplementary Fig. 18). This can be explained by the Wnt-surrogate concentration being highest in direct vicinity of the DNA microbeads, in agreement with the computer simulations of the off-centred microbead inclusion (Supplementary Fig. 15). While the exact shape of the induced RPE was found to vary between organoids, likely owing to inter-organoid heterogeneity regarding the distribution of cells susceptible to the inductive Wnt signal, RPE was induced both in shapes that mimic the in vivo condition as well as in shapes that were entirely different from it. The presented technology thus gives the user control over the differentiation pattern.

To showcase the possibility of adding multiple functional moieties, we added a cholesterol group to the DNA microbeads. The Wnt-DNA microbead design was thus changed to where the Wnt-surrogate is released after DNA microbead breakdown while attached to a cholesterol-modified DNA-Y-motif (Wnt-cholesterol-DNA microbeads; Supplementary Fig. 19a). As such, it is possible to release Wnt-surrogate and remove the DNA microbeads in a single step. This did not result in a further restriction of the RPE differentiation area by cholesterol-mediated reduction of diffusivity. Nonetheless, this highlights that a dual cargo release is feasible with the DNA microbead technology (Requirement (v)). Of note, Wnt-DNA microbead microinjected organoids sometimes developed tiny hubs of RPE without UV light-triggered Wnt-surrogate release. This might be due to Wnt-surrogate acting upon the cells directly adjacent to the Wnt-DNA microbeads even without release, although a minor non-detectable DNA microbead degradation cannot be ruled out as a cause.

## Conclusion

This work demonstrates that cell-sized, stiffness-adaptable DNA microbeads can be integrated into organoids via microinjection and that their cargo can be released non-invasively by light. The technology allows for spatial and temporal user control in the bioengineering of organoids with internally provided morphogens throughout their development. While this work presents a first proof-of-principle application of this mechanism using proteins, on the same principle, delivery of any click-chemistry addressable molecule into tissues is feasible. The presented technology addresses the need for implementation of morphogen sources into 3D organoid cell cultures of any developmental stage and opens up their intricate interior microarchitecture to precise bioengineering efforts.

## Methods

### Design and handling of DNA sequences

The sequences used to prepare the DNA-Y-motifs YA and YB as well as the DNA linker were adapted from previous publications^29,40^. DNA strands were purchased either from Integrated DNA Technologies (unmodified DNA, purification: standard desalting) or Biomers (modified DNA, purification: HPLC). All DNA, apart from fluorophore-labelled strands, was diluted in 10 mM Tris (pH 8) and 1 mM EDTA (Sigma Life Science) to yield 800 μM stock solutions. Fluorophore-labelled strands were diluted in MilliQ water to yield 800 μM stock solutions. All utilized DNA sequences are listed in Supplementary Table 7. The DNA stock solutions were stored at −20 °C.

### Preparation of Y-motif DNA

The DNA-Y-motifs (YA and YB) needed to form the DNA microbeads were produced via thermal annealing of the three respective single-stranded DNA strands YA-1, YA-2 and YA-3 for YA, or YB-1, YB-2 and YB-3 for YB. The strands were mixed at equimolar ratios to yield a final concentration of the resulting Y-motifs of 150 µM. In all experiments, 4 mol% of Cyanine-3 (Cy3)-labelled YB-1 strand was added to the YB mixture to allow for fluorescence microscopy of the resulting DNA microbeads. The Y-motifs were annealed in a solution containing 1× PBS (Gibco). Annealing was conducted in a thermal cycler (BioRad) by heating the samples to 85 °C for 3 min and subsequently cooling the sample to 20 °C using an increment rate of −0.1 °C s^−1^.

### Formation of DNA microbeads

DNA microbeads were created in a templated manner after encapsulation of the gelation solution into water-in-oil droplets. To form the DNA microbeads, the annealed Y-motifs YA and YB were mixed at equimolar ratios (20 µM, 25 µM or 30 µM) in a solution containing 1× PBS. The DNA linker strand was then added to the solution in 3× excess to the Y-motifs (for example, 30 µM Y-motifs + 90 µM DNA linker). Immediately after the addition of the DNA linker, the mixture was added on top of an oil phase containing 2 wt% perfluoropolyether–polyethylene glycol (PFPE–PEG, RAN Biotechnologies) dissolved in HFE-7500 (Iolitex Ionic Liquids Technologies) at a ratio of 1:3 aqueous phase to oil phase (for example, 50 µl aqueous solution and 150 µl oil mixture) and the reaction tube with the mixture flicked with a finger 8× to create an emulsion. The resulting water-in-oil droplet emulsion was incubated at 22 °C room temperature for 72 h to ensure full gelation of the DNA microbeads. After this, the DNA microbeads were released by breaking the water-in-oil emulsion. To release the microbeads, a 1× PBS solution was added on top of the droplet emulsion. Subsequently, the emulsion was destabilized by adding the surfactant 1*H*,1*H*,2*H*,2*H*-perfluoro-1-octanol (Merck) on top of the buffer. This mix was incubated for 30 min before the resulting aqueous phase containing the DNA microbeads was taken off and transferred to a separate reaction tube. The DNA microbeads were stored at 5 °C before their use and prepared fresh for each experiment. DNA microbead components and their concentrations for all microbeads used in this study are detailed in Supplementary Table 8.

### Real-time deformability cytometry

RT-DC was performed using an AcCellerator (Zellmechanik Dresden) mounted on an inverted AxioObserver microscope (Carl Zeiss AG) equipped with a 20×/0.4 Ph2 Plan-NeoFluar objective (Carl Zeiss AG). Images were acquired using a high-speed CMOS camera (MC1362, Microtron).

To measure the DNA microbeads, a suspension of microbeads (100 µl) was strained through a 20 µm EASYstrainer filter (Greiner Bio-One) and pelleted in a reaction tube by spinning them down for 2 min with a C1008-GE myFUGE mini centrifuge (Benchmark Scientific). The supernatant (80 µl) was then taken off and discarded, and the remaining pellet of DNA microbeads was resuspended in 150 µl of CellCarrierB (Zellmechanik Dresden). The resuspended microbeads were then aspirated into a 1 ml glass syringe with a PEEK tubing connector and PTFE plunger (SETonic) mounted on a syringe pump system (NemeSys, Cetoni). The DNA microbead-CellCarrierB solution was then injected into a Flic20 microfluidic chip (Zellmechanik Dresden) using PTFE tubing (S1810-12, Bola). Through a second 1 ml glass syringe, CellCarrierB was injected into the Flic20 microfluidic chip as sheath flow for the RT-DC experiment. For all samples, measurements at 0.04 µl s^−1^ total flow rate (ratio of sheath-to-sample flow 3:1) were run for a duration of at least 900 s each. The measurement software ShapeIn (version 2.2.2.4, Zellmechanik Dresden) was used to detect the DNA microbeads in real time. The pixel size was adjusted to 0.68 µm px^−1^, fitting the utilized 20×/0.4 Ph2 objective and all DNA microbeads imaged at the rear part of the flow channel ensuring regular deformation of each microbead. For each condition, triplicates were measured. Measurements of the DNA microbeads containing Wnt-surrogate were conducted in the same way, following an overnight incubation of the DNA microbeads with a Wnt-surrogate-modified DNA linker (see section ‘Formation of DNA microbeads with PC Wnt-surrogate’) and three washing steps using 1× PBS. Before the overnight incubation, the DNA microbeads were likewise filtered through a 20 µm EASYstrainer filter (Greiner Bio-One).

The same workflow was applied to dissociated medaka RO cells. In preparation for RT-DC, medaka RO were cultivated as described in ‘Generation of medaka-derived RO’ until late day 1. Forty-eight organoids per experiment were then pooled into 2 ml tubes and washed multiple times with 1× PBS. Dissociation was performed by incubation in dissociation solution (1:1 dilution of 2.5% Trypsin (Gibco, catalogue number 15090046) and 1 U ml^−1^ Dispase (Stemcell Technologies, catalogue number 15569185)) for 10 min under gentle shaking and occasional gentle pipetting at 28 °C. Trypsin was quenched by diluting the dissociation solution 1:2 in 50% FBS containing 1× PBS solution. Single cells were spun down at 200 × *g* at room temperature for 3 min, the supernatant was aspirated, and cells were resuspended in 150 µl CellCarrierB. The cells were likewise measured as triplicates (48 organoids each) resulting from independent sets of organoids for each measurement.

Following RT-DC, the utilized microfluidic chips were flushed with a fluorescein-MilliQ water solution and z-stacks of the flow channels acquired with an LSM 900 Zeiss confocal fluorescence microscope (Carl Zeiss AG). For each z-stack, the pinhole size was set to one Airy unit and a Plan-Apochromat 20×/0.8 Air M27 objective was used. The median width of each flow channel was then calculated from the z-stack using a custom Python script and the RT-DC data corrected accounting for the width of the respective flow channel.

The analysis software Shape-Out (version 2.10.0, Zellmechanik Dresden) was then used for data analysis. All samples were gated for porosity (1.0–1.2) and size (65–160 µm^2^). Statistical analysis based on a linear mixed model (R-lme4) as implemented in Shape-Out (version 2.10.0, Zellmechanik Dresden^53^), calculation of Young’s moduli, deformation and volume as well as preparation of the data for contour and violin plots were all carried out using Shape-Out (version 2.10.0, Zellmechanik Dresden). The linear mixed model was run without adjustments. *P*-value calculations to determine statistical significance are based on analysis of variance (ANOVA) test to correctly analyse the data as derived from RT-DC measurements^53^. Plots for the volume, deformation and Young’s modulus were created using OriginPro 2021, Update 6 (OriginLab Corporation).

### Formation of PC DNA microbeads and quantification of DNA microbead disassembly using light

PC DNA microbeads were formed in the same way as detailed above. However, 60% of the utilized linkers contained a PC moiety in the centre of the DNA linker sequence (PC linker; for details, see Supplementary Table 7). In triplicates, five PC DNA microbeads per sample were analysed to quantify the breakdown of the DNA microbeads following exposure to 405 nm light. The microbeads were chosen to be 50 µm in diameter and imaged using 5× digital zoom. The frame time was set to 148.95 ms and the pixel size of the acquired image to 256 × 256 px. To break down the DNA microbeads, the laser power of a 405 nm confocal laser (5 mW maximum power) was set to 10% and the microbeads were continuously irradiated for 60 s, resulting in their disassembly. In addition, DNA microbeads without PC linker (five per replicate with three replicates total) were treated in the same way as above as a negative control. Analysis of the disassembly was then performed in Fiji (NIH^54^). For this, the mean fluorescence signal across the irradiated images was acquired and the data normalized to the first frame of each video. The data were plotted using OriginPro 2021, Update 6 (OriginLab Corporation).

### Conjugation of Wnt-surrogate proteins to DNA linkers

WNT-surrogate-Fc fusion protein (Wnt-surrogate; ImmunoPrecise Antibodies; catalogue number N001, lot 5696, 6384, 7134, 7568) was dialysed against 25 mM HEPES and 500 mM NaCl buffer at pH = 8.2 using ZelluTrans/Roth Mini Dialyzer tubes MD300 (12–14 kDa, Carl Roth). Dialysis was conducted at 4 °C for 36 h with hourly buffer changes during the day and a long incubation overnight to remove Tris from the buffer solution. Modification of the Wnt-surrogate with an azide moiety was achieved using an azidobutyric-NHS ester (Lumiprobe) according to the manufacturer’s recommendations. Further, modification of the Wnt-surrogate with Alexa Fluor 647 (Wnt-AF647) was achieved by adding an NHS-modified Alexa Fluor 647 ester (AF647N-NHS, Lumiprobe) simultaneously to the azidobutyric-NHS ester in accordance with the manufacturer’s recommendations.

The resulting solution was then again dialysed against 25 mM HEPES and 500 mM NaCl buffer at pH = 8.2 in the same way as before to remove any unreacted NHS esters. DBCO-modified DNA linker strands (PC or non-PC; Supplementary Table 7) were then added to the azide-modified (azide/AF647-modified) Wnt-surrogate in a 1:1 ratio and incubated to react for 76 h, yielding a final concentration of 8 µM Wnt-surrogate-modified (Wnt-AF647-surrogate-modified) DNA linker.

### Formation of DNA microbeads with PC Wnt-surrogate

After a DNA microbead suspension was passed through a 20 µm filter, 30 µl of this DNA microbead suspension was pelleted using a C1008-GE myFUGE mini centrifuge (Benchmark Scientific) for 2 min. Then, 20 µl of the supernatant was removed to leave 10 µl of the DNA microbead pellet in the reaction tube. To achieve the incorporation of DNA linker with PC Wnt-surrogate, the DNA microbead pellet was resuspended with 10 µl of PC Wnt-surrogate-modified DNA linker (8 µM), yielding a final concentration of 4 µM modified linker. The mixture was incubated overnight, after which the microbeads were washed three times using 100 µl of a 1× PBS solution to remove non-incorporated DNA linkers and proteins, yielding a final volume of 10–15 µl of modified DNA microbeads after removal of the washing solution after centrifugation. Formation of DNA microbeads with Alexa Fluor 647-labelled Wnt-surrogate was conducted in the same way using Wnt-AF647-modified DNA linkers. Note that substantially less than 1 µl of the final DNA microbead suspension is used for the microinjection of up to 50 organoids. The volume produced this way is thus sufficient for the microinjection of more than 500 organoids.

### Quantification of the release of Alexa Fluor 647-modified Wnt-surrogate (Wnt-AF647) from DNA microbeads

To quantify the release of Wnt-AF647 from the DNA microbeads, the microbeads (*n* = 5) were illuminated with a 405 nm laser at 10% power (5 mW maximum power) and imaged for 180 s until the Wnt-AF647 signal was depleted. Irradiation of the DNA microbeads with the 405 nm laser started 20 s after the start of the imaging. The frame time was set to 148.95 ms and the pixel size of the acquired image to 256 × 256 px during imaging. The mean fluorescence signal of the Alexa Fluor 647 dye within the DNA microbeads was then measured using the circle tool in Fiji (NIH^54^) across all frames. All data were normalized to the mean fluorescence detected in the first frame of each video and plotted using OriginPro 2021, Update 6 (OriginLab Corporation).

### Fish husbandry and maintenance

Medaka (*O. latipes*) stocks were maintained according to the local animal welfare standards (Tierschutzgesetz §11, Abs. 1, Nr. 1, husbandry permit AZ35-9185.64/BH, line generation permit number 35-9185.81/G-145/15 Wittbrodt). Fish are kept as closed stocks in constantly recirculating systems at 28 °C with a 14 h light/10 h dark cycle. The following medaka lines were used in this study: Cab strain as a wild type^55^ and *Atoh7::EGFP*^56^.

### Generation of medaka-derived RO

Medaka-derived RO were generated as previously described^3^ with slight modifications to the procedure. In brief, medaka primary embryonic pluripotent cells were isolated from whole blastula-stage (6 h post fertilization) embryos^44^ and resuspended in modified differentiation media (DMEM/F12 (Dulbecco’s modified Eagle medium/Nutrient Mixture F-12, Gibco, catalogue number 21041025), 5% KSR (Gibco, catalogue number 10828028), 0.1 mM non-essential amino acids, 0.1 mM sodium pyruvate, 0.1 mM β-mercaptoethanol, 20 mM HEPES pH = 7.4, 100 U ml^−1^ penicillin–streptomycin). The cell suspension was seeded in densities of 1,500 cells per organoid (approximately 15 cells per µl) for standard-sized organoids and 500 cells per organoid for small organoids in 100 µl per well in a low-binding, U-bottom-shaped 96-well plate (Nunclon Sphera U-Shaped Bottom Microplate, Thermo Fisher Scientific, catalogue number 174925) and centrifuged (180 × *g*, 3 min at room temperature) to speed up cell aggregation. At day 1, aggregates were transferred to fresh differentiation media and Matrigel (Corning, catalogue number 356230) was added to the media for 9 h to a final concentration of 2%. From day 2 onwards, RO were kept in maturation media (DMEM/F12 supplemented with 10% FBS (Sigma-Aldrich, catalogue number 12103C), 1× N2 supplement (Gibco, catalogue number 17502048), 1 mM taurine (Sigma-Aldrich, catalogue number T8691), 20 mM HEPES pH = 7.4, 100 U ml^−1^ penicillin–streptomycin). For the analysis of the spatial correlation between the DNA microbeads’ position and the induced RPE differentiation, organoids were kept in differentiation media for the whole duration of organoid culture. RO thus developed less RPE after induction (alongside generally being smaller). This enabled a more precise investigation of the spatial relationship of the DNA microbead position and the emerging RPE differentiation pattern after DNA microbead-mediated Wnt-surrogate release at day 1.

RO were either derived from embryos of wild-type Cab strain only (Figs. 2b and 3, and Supplementary Fig. 12) or mixed with blastomeres of blastula-stage embryos of the Atoh7::EGFP transgenic line (outcrossed to Cab) in a 4:1 ratio. Mixing primary pluripotent embryonic stem cells from wild-type and transgenic embryos in this ratio ensured that only a fraction of retinal ganglion cells was being reported for. This facilitated the identification of qualitative differences in cell numbers and distribution within individual organoids owing to reduced clustering of reporter cells. In this way, the labelled retinal ganglion cells were used as a proxy for the overall formation of neuroretina in the organoids.

### RO microinjection

For microinjection, day 1 RO were washed 3 times after 9 h of Matrigel incubation, transferred onto Parafilm (Thermo Fisher Scientific, catalogue number 13-374-10) and lined up against the edge of a square coverslip (24 × 24 mm) in differentiation media. Borosilicate micropipettes (1 mm OD × 0.58 mm ID × 100 mm L; Warner Instruments, catalogue number 30-0016) were pulled on a Flaming/Brown micropipette puller P-97 (Sutter Instruments) with the following settings: heat 505, pull 25, velocity 250, time 10, 1 cycle. The microinjection was performed with a CellTram 4m oil microinjector (Eppendorf AG) and a standard manual micromanipulator under an epifluorescence stereomicroscope (Olympus MVX10; MV PLAPO 1× objective) to visualize Cy3 fluorescently labelled DNA microbeads during microinjection. Note that all DNA microbead suspensions used for microinjection into RO were passed through a 20 µm filter before microinjection.

For UV light-triggered release of the DNA microbead’s cargo or disassembly of DNA microbeads themselves in live RO, organoids kept in 100 µl differentiation media on a culture dish were exposed for 60 s at a 1 cm distance to Leica EL6000 (100% intensity; Lamp HXP-R120W/45C VIS, power input 120 W, Osram Licht AG). Analysis of the disassembly (Supplementary Fig. 6) was then performed in Fiji (NIH^54^). For this, the mean fluorescence signal across a region of interest (ROI) of the DNA microbead position within the images was acquired and the data normalized to the first frame of the time-lapse imaged RO.

Wnt-surrogate release from DNA microbeads was conducted 2 h post microinjection on day 1 of RO culture, since Wnt-surrogate-mediated induction of RPE was found to be only possible on late day 1.

### Embryo microinjection

Stage 20 (1 day post fertilization) embryos^44^ were dechorionated using hatching enzyme, washed and kept in 100 U ml^−1^ penicillin–streptomycin containing ERM (17 mM NaCl, 40 mM KCl, 0.27 mM CaCl_2_, 0.66 mM MgSO_4_, 17 mM HEPES). Embryos were transferred onto a 1% agarose mould^57^, oriented heads down for microinjection and punctured at the vegetal pole. Microinjected embryos were re-cultured on glass ware in 100 U ml^−1^ penicillin–streptomycin containing ERM until hatchling stage (s40 (ref. ^44^)) with daily assessment of their gross morphology by stereomicroscopy.

### Radial diffusion analysis of Cy3-labelled DNA-Y-motif and Wnt-AF647 in small RO

For the radial diffusion analysis of the DNA-Y-motif and Wnt-AF647, the pixels with intensities above the 0.98 and 0.99 intensity quantiles in the initial images, respectively, were averaged to obtain the centre of mass positions (COM). For the Wnt-AF647, the sum projection was considered to average over a height of 30 µm.

Around the COM, the image intensities were radially averaged in azimuthal sections of 60° (Fig. 3g,f). For the Wnt-AF647, the boundary of the inclusion region in the individual sections was determined as the maximum radius with a half-maximum intensity in the Wnt-AF647 channel. For the DNA-Y-motif, the imaging plane barely touched the microinjection region and thus the inner boundary is assumed to lie at radius 0. The outer boundary in the sections was determined as the averaged boundary from manual segmentation (Wnt-AF647; Fig. 3e), or the maximum radius with an averaged half-maximum intensity as measured from the plasma membrane staining (DNA-Y-motif; Fig. 3d). For each section, the radially averaged concentration profiles between the inclusion and the organoid boundary were rescaled to the interval (0,1) and then all datasets were spatially averaged with a moving average approach with a 10 times smaller resolution as the coarsest resolution in the sections. Within these averaging intervals, the standard deviation was calculated to obtain the error bands.

### Statistics and reproducibility

Statistical analysis was conducted either using a linear mixed model approach, deriving a *P* value using ANOVA test (according to the RT-DC workflow as published^53^ and implemented in the analysis software Shape-Out (version 2.10.0, Zellmechanik Dresden; for details, see section ‘Real-time deformability cytometry’)), or using two-tailed Student’s *t*-test with unequal variance (calculation of significant differences in Fig. 4). In all cases, *P* values < 0.05 were considered statistically significant. Sample sizes and the data presented were chosen to reflect representative fractions of the overall data. No statistical method was used to predetermine sample size. No data were excluded from the analyses. The experiments were not randomized and the investigators were not blinded to allocation during experiments and outcome assessment.

### Reporting summary

Further information on research design is available in the Nature Portfolio Reporting Summary linked to this article.

## Online content

Any methods, additional references, Nature Portfolio reporting summaries, source data, extended data, supplementary information, acknowledgements, peer review information; details of author contributions and competing interests; and statements of data and code availability are available at 10.1038/s41565-024-01779-y.

## Supplementary information

Supplementary Information Supplementary Tables 1–8, Figs. 1–20, Videos 1–3, Note 1, note references, methods and methods references.

Reporting Summary

Supplementary Video 1 Diffusion dynamics of Cy3-DNA-Y-motif after DNA microbead breakdown in live small RO.

Supplementary Video 2 Diffusion dynamics of Wnt-AF647 after release from DNA microbeads in live small RO.

Supplementary Video 3 Spatial correlation between DNA microbead position over time and Wnt-surrogate-release induced RPE differentiation pattern in RO.

## Data Availability

The data that support the findings of this study are available on HeiData, the Open Research Data institutional repository for Heidelberg University, with the identifier 10.11588/data/T87EPK.

## Code availability

The code that was used to analyse the data and solve the finite element model is available on HeiData, the Open Research Data institutional repository for Heidelberg University, with the identifier 10.11588/data/T87EPK.
