# Supplementary material for: DNA microbeads for spatio-temporally controlled morphogen release within organoids
Source: Nat Nanotechnol. 2024 Sep 9;19(12):1849–57. doi: 10.1038/s41565-024-01779-y (PMC11638066; doi:10.1038/s41565-024-01779-y)
Supplement: Supplementary file 1 — Supplementary Tables 1–8, Figs. 1–20, Videos 1–3, Note 1, note references, methods and methods references. [file 41565_2024_1779_MOESM1_ESM.pdf]

nature nanotechnology

Supplementary information

https://doi.org/10.1038/s41565-024-01779-y

DNA microbeads for spatio-temporally controlled morphogen release within organoids

In the format provided by the authors and unedited

Table of Contents

Supplementary Tables* *3
- **Supplementary Table 1 .... 3**
- **Supplementary Table 2 .... 4**
- **Supplementary Table 3 .... 5**
- **Supplementary Table 4 .... 6**
- **Supplementary Table 5 .... 6**
- **Supplementary Table 6 .... 6**
- **Supplementary Table 7 .... 7**
- **Supplementary Table 8 .... 8**

Supplementary Figures* *9
- **Supplementary Figure 1 .... 9**
- **Supplementary Figure 2 .... 10**
- **Supplementary Figure 3 .... 11**
- **Supplementary Figure 4 .... 12**
- **Supplementary Figure 5 .... 13**
- **Supplementary Figure 6 .... 14**
- **Supplementary Figure 7 .... 15**
- **Supplementary Figure 8 .... 16**
- **Supplementary Figure 9 .... 18**
- **Supplementary Figure 10 .... 19**
- **Supplementary Figure 11 .... 20**
- **Supplementary Figure 12 .... 21**
- **Supplementary Figure 13 .... 22**
- **Supplementary Figure 14 .... 23**
- **Supplementary Figure 15 .... 24**
- **Supplementary Figure 16 .... 25**
- **Supplementary Figure 17 .... 26**
- **Supplementary Figure 18 .... 27**
- **Supplementary Figure 19 .... 28**
- **Supplementary Figure 20 .... 29**

Supplementary Videos* *30

Supplementary Video 1 .... **30**

Supplementary Video 2 .... **30**

Supplementary Video 3 .... **30**

Supplementary Notes ***31***

Supplementary Note 1 .... **31**

Diffusion-degradation model for externally supplied morphogens 31

Modelling morphogen release through optical hydrogel disassembly and through Wnt-surrogate cleavage 32

Modelling concentrations for off-centered morphogen supply 33

Details of the finite element implementation of the model 33

Supplementary Note References ***35***

Supplementary Methods ***36***

Confocal fluorescence microscopy of DNA microbeads 36

Sodium dodecyl sulfate polyacrylamide gel electrophoresis 36

Formation of DNA microbeads with photocleavable Wnt-modified Y-motifs 36

Formation of DNA microbeads with photocleavable 5-fluorescein-amidite (5-FAM) 37

Quantification of the release of 5-fluorescein-amidite (5-FAM) from DNA microbeads 37

Sample preparation and workflow for microindentation 37

Fluorescence recovery after photobleaching 38

Quantification of Cy3-labeled Y-motif uptake into unlabeled DNA microbeads 38

Fluorescent labeling and imaging of medaka retinal organoids and embryos 38

Quantification of area of retinal pigmented epithelium and retinal ganglion cell numbers 40

Supplementary Methods References ***41***

Supplementary Tables

Supplementary Table 1

Supplementary Table 1: Volumes of the DNA microbeads and medaka retinal organoid cells as measured via real-time deformability cytometry.

Repetition	N	Mean volume [µm^3]	Standard deviation [µm^3]	Triplicate mean [µm^3]	Standard deviation [µm^3]	Standard error of mean [µm^3]
**20 µM DNA microbeads**						
**1**	7346	477.9	180.7	473.7	303.8	3.2
**2**	8127	485	178.7			
**3**	16555	458.1	166.4			
**25 µM DNA microbeads**						
**1**	11573	479.1	180.9	540.4	354.4	3.1
**2**	10271	566.7	213.4			
**3**	19293	575.3	217.5			
**30 µM DNA microbeads**						
**1**	11627	582.4	220.7	553.9	366.2	3.3
**2**	12900	547.9	208.7			
**3**	13727	531.3	204.5			
**Medaka retinal organoid cells**						
**1**	7873	564.3	168	555.3	290	3.2
**2**	6675	547.8	154.6			
**3**	11305	553.9	178.8			

Supplementary Table 2

Supplementary Table 2: Deformation of the DNA microbeads and medaka retinal organoid cells as measured via real-time deformability cytometry.

Repetition	N	Mean deformation	Standard deviation	Triplicate mean	Standard deviation	Standard error of mean
**20 µM DNA microbeads**						
**1**	7346	0.081	0.016	0.089	0.035	0.00034
**2**	8127	0.089	0.021			
**3**	16555	0.097	0.023			
**25 µM DNA microbeads**						
**1**	11573	0.098	0.02	0.084	0.032	0.00028
**2**	10271	0.08	0.018			
**3**	19293	0.074	0.017			
**30 µM DNA microbeads**						
**1**	11627	0.068	0.017	0.075	0.03	0.00027
**2**	12900	0.08	0.018			
**3**	13727	0.077	0.017			
**Medaka retinal organoid cells**						
**1**	7873	0.082	0.027	0.076	0.047	0.00051
**2**	6675	0.071	0.025			
**3**	11305	0.075	0.03			

Supplementary Table 3

Supplementary Table 3: Young's moduli of the DNA microbeads and medaka retinal organoid cells as calculated from real-time deformability cytometry data.

<table>
<tr><th>Repetition</th><th>N</th><th>Mean Young’s modulus [kPa]</th><th>Standard deviation [kPa]</th><th>Triplicate mean [kPa]</th><th>Standard deviation [kPa]</th><th>Standard error of mean [kPa]</th></tr>
<tr><td colspan="7">20 µM DNA microbeads</td></tr>
<tr><td>1</td><td>7346</td><td>0.71</td><td>0.11</td><td rowspan="3">0.7</td><td rowspan="3">0.19</td><td rowspan="3">0.0019</td></tr>
<tr><td>2</td><td>8127</td><td>0.72</td><td>0.11</td></tr>
<tr><td>3</td><td>16555</td><td>0.68</td><td>0.1</td></tr>
<tr><td colspan="7">25 µM DNA microbeads</td></tr>
<tr><td>1</td><td>11573</td><td>0.78</td><td>0.12</td><td rowspan="3">0.79</td><td rowspan="3">0.26</td><td rowspan="3">0.0019</td></tr>
<tr><td>2</td><td>10271</td><td>0.78</td><td>0.13</td></tr>
<tr><td>3</td><td>19293</td><td>0.81</td><td>0.12</td></tr>
<tr><td colspan="7">30 µM DNA microbeads</td></tr>
<tr><td>1</td><td>11627</td><td>0.96</td><td>0.15</td><td rowspan="3">0.89</td><td rowspan="3">0.26</td><td rowspan="3">0.0024</td></tr>
<tr><td>2</td><td>12900</td><td>0.87</td><td>0.17</td></tr>
<tr><td>3</td><td>13727</td><td>0.87</td><td>0.14</td></tr>
<tr><td colspan="7">Medaka retinal organoid cells</td></tr>
<tr><td>1</td><td>7873</td><td>0.81</td><td>0.22</td><td rowspan="3">0.88</td><td rowspan="3">0.48</td><td rowspan="3">0.0049</td></tr>
<tr><td>2</td><td>6675</td><td>0.84</td><td>0.19</td></tr>
<tr><td>3</td><td>11305</td><td>0.99</td><td>0.38</td></tr>
</table>

Supplementary Table 4

Supplementary Table 4: Volumes of the Wnt-modified DNA microbeads as measured via real-time deformability cytometry.

Repetition	N	Mean volume [µm^3]	Standard deviation [µm^3]	Triplicate mean [µm^3]	Standard deviation [µm^3]	Standard error of mean [µm^3]
**30 µM Wnt-modified DNA microbeads**						
**1**	8704	608.7	228.9	596.5	321.8	4.2
**2**	4303	584.3	226.2			

Supplementary Table 5

Supplementary Table 5: Deformation of the Wnt-modified DNA microbeads as measured via real-time deformability cytometry.

Repetition	N	Mean deformation	Standard deviation	Triplicate mean	Standard deviation	Standard error of mean
**30 µM Wnt-modified DNA microbeads**						
**1**	8704	0.062	0.015	0.06	0.021	0.00027
**2**	4303	0.058	0.014			

Supplementary Table 6

Supplementary Table 6: Young's moduli of the Wnt-modified DNA microbeads as measured via real-time deformability cytometry.

Repetition	N	Mean Young's modulus [kPa]	Standard deviation [kPa]	Triplicate mean [kPa]	Standard deviation [kPa]	Standard error of mean [kPa]
**30 µM Wnt-modified DNA microbeads**						
**1**	8704	0.95	0.16	0.96	0.24	0.0031
**2**	4303	0.96	0.17			

Supplementary Table 7

Supplementary Table 7: DNA sequences used for this study. The fluorescent label cyanine 3 is abbreviated as Cy3, the photocleavable group is denoted as BMN. Cholesterol is abbreviated as Chol.

Name	DNA sequence 5' - 3'
YA-1	GACCAACACCAGTGAGGACGGAAGTTTGTCGTAGCATCGCACC
YA-1-Chol	GACCAACACCAGTGAGGACGGAAGTTTGTCGTAGCATCGCACCTTT-Chol
YA-2	GACCAACACCAACCACGCCTGTCCATTACTTCCGTCCTCACTG
YA-3	GACCAACACGGTGCGATGCTACGACTTTGGACAGGCGTGGTTG
YB-1	CAGTGAGGACGGAAGTTTGTCGTAGCATCGCACCCGACAGGAA
YB-1-Cy3	Cy3-CAGTGAGGACGGAAGTTTGTCGTAGCATCGCACCCGACAGGAA
YB-2	CAACCACGCCTGTCCATTACTTCCGTCCTCACTGCGACAGGAA
YB-3	GGTGCGATGCTACGACTTTGGACAGGCGTGGTTGCGACAGGAA
linker	GTGTTGGTCTTCCTGTCG
PC-linker	GTGTTGGTC-BMN-TTCCTGTCG
DBCO-linker	DBCO-TTTGTGTTGGTCTTCCTGTCG
DBCO-PC-linker	DBCO-BMN-GTGTTGGTCTTCCTGTCG

Supplementary Table 8

Supplementary Table 8: DNA type and concentrations used for the formation of DNA microbeads for this study. Photocleavability is denoted as PC, while cholesterol is abbreviated as Chol.

**DNA microbead type**	**Y-motif concentration [µM]**	**DNA linker concentration [µM]**	**DNA type added after formation [µM]**
20 µM	20 YA, 20 YB	60	-
25 µM	25 YA, 25 YB	75	-
30 µM	30 YA, 30 YB	90	-
30 µM PC	30 YA, 30 YB	54 PC-linker<br>36 DNA linker	-
30 µM Wnt-surrogate modified	30 YA, 30 YB	90	Wnt-modified DNA linker (4)
30 µM Wnt-surrogate/cholesterol-modified	30 YA, 30 YB	54 PC-linker<br>36 DNA linker	YA-Chol (1.3)<br>Wnt-modified DNA linker (4)

Supplementary Figures

Supplementary Figure 1

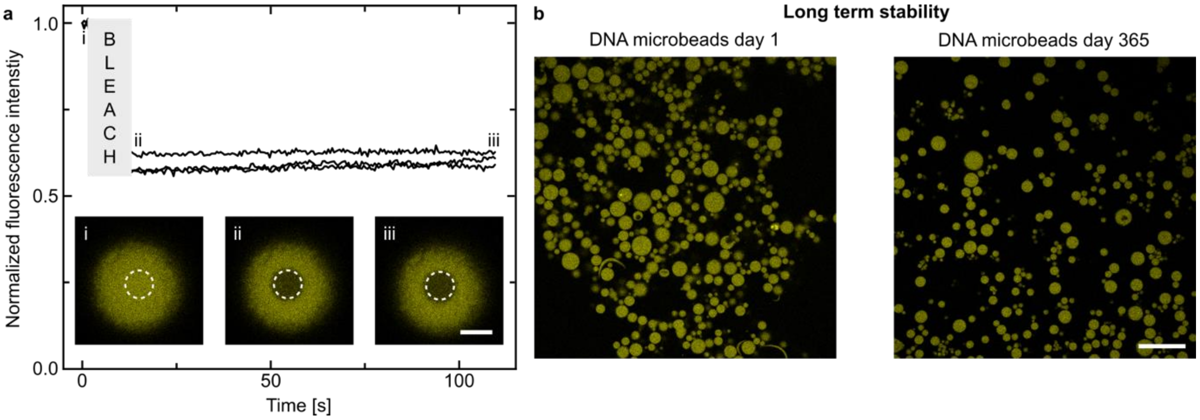

Supplementary Fig. 1: Network stability of the DNA microbeads. a) Graph showing the fluorescence recovery after photobleaching behavior of three separate DNA microbeads. Inserted confocal micrographs (λ_{ex} = 561 nm, Cy3-labeled Y-motif, yellow) before (i) and after bleaching (ii, iii). Scale bar: 10 µm. b) Confocal microscopy images (λ_{ex} = 561 nm, Cy3-labeled Y-motif, yellow) of DNA microbeads at day 1 and day 365 post formation. Scale bar: 100 µm.

Supplementary Figure 2

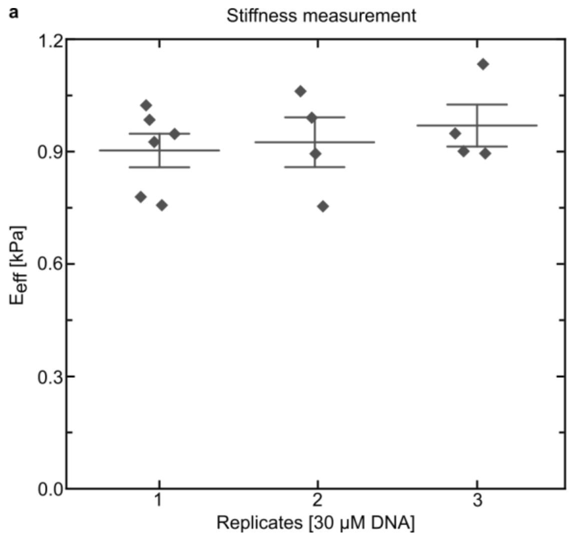

Supplementary Fig. 2: Effective elastic modulus of DNA microbeads at 30 µM DNA concentration in 1x PBS as measured by microindentation. For each replicate (n = 3), at least four microbeads were measured. The mean ± standard deviation and all individual data points of each replicate are shown.

Supplementary Figure 3

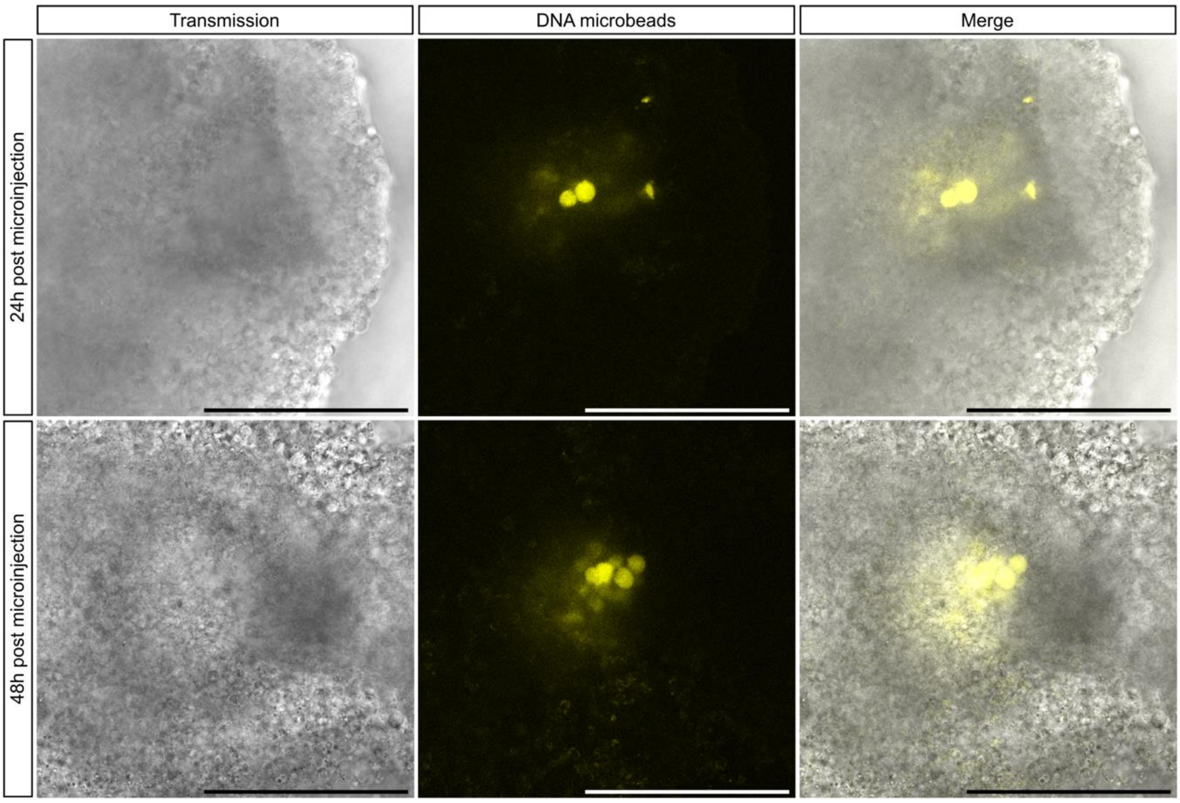

Supplementary Fig. 3: DNA microbeads maintain structural integrity post microinjection into RO and sustain it. Representative confocal images of chemically fixed day 3 and day 4 RO after microinjection with DNA microbeads on day 2. Scale bars: 100 µm.

Supplementary Figure 4

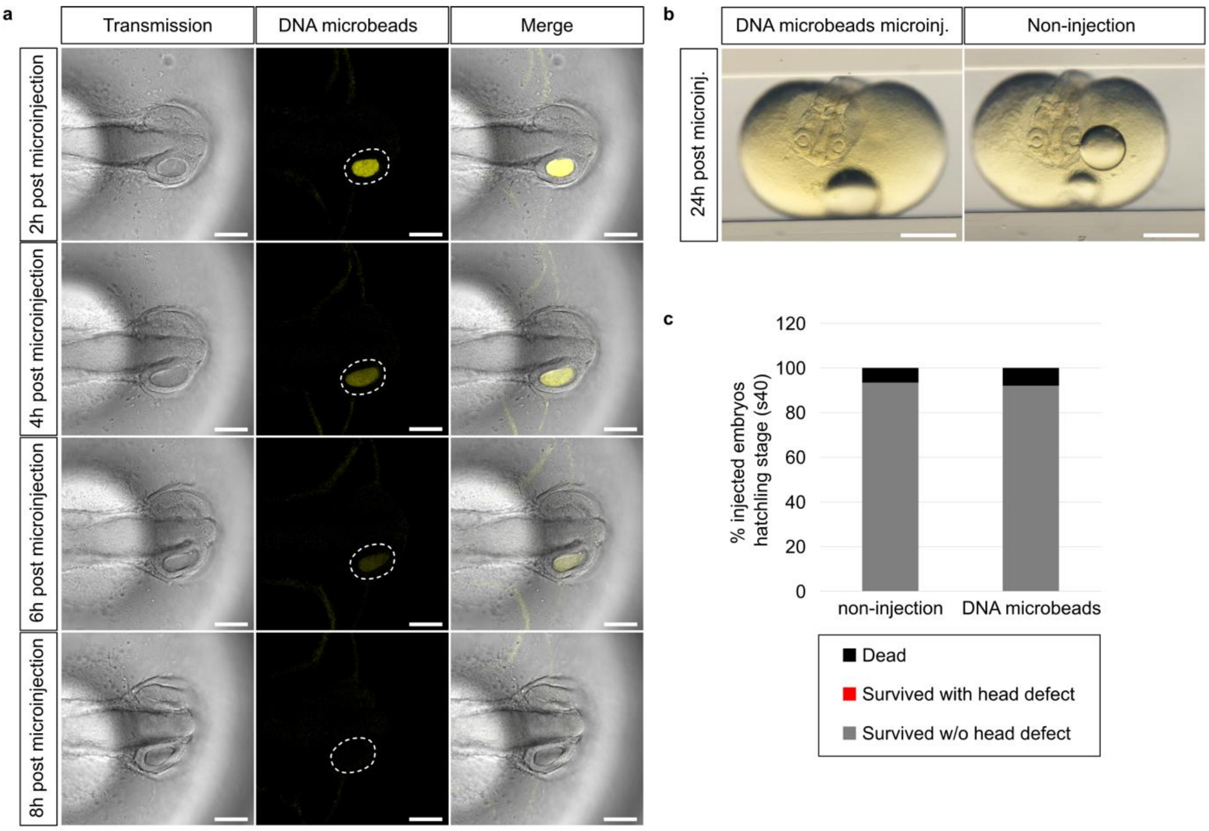

Supplementary Fig. 4: DNA microbeads dynamics in the developing medaka retina. a) Representative transmission images of live stage 20 medaka embryo 2, 4, 6 and 8 h after DNA microbeads microinjection into its optic vesicle. The DNA microbead channel was subjected to a median filter with 1.5 pixel radius to reduce the autofluorescence of the medaka embryo yolk. Scale bars: 100 µm. b) Stereomicroscopic brightfield images of the embryo shown in a (DNA microbeads microinj.) and its respective uninjected control (non-injection). Scale bars: 500 µm. c) Quantification of survival and gross developmental head defects in stage 40 medaka hatchlings with (DNA microbeads; n = 25 embryos) and without (non-injection; n = 30 embryos) DNA microbeads microinjection into their optic vesicles at stage 20.

Supplementary Figure 5

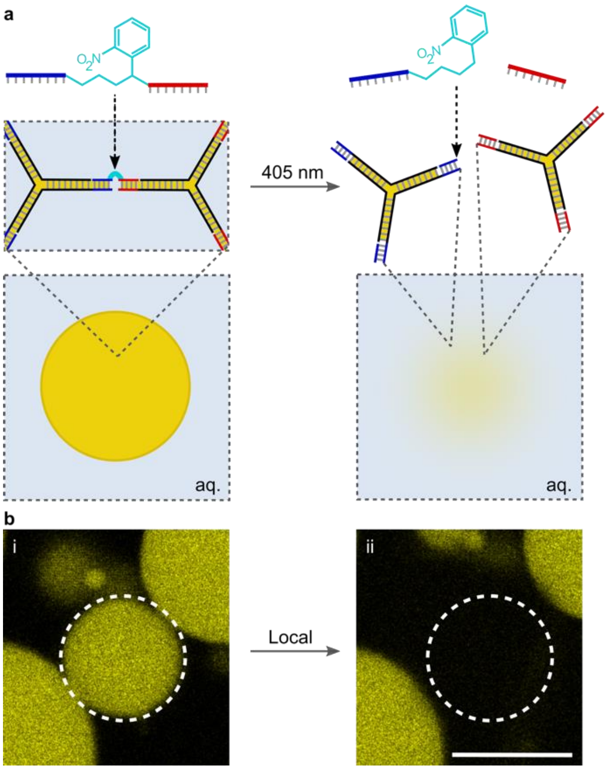

Supplementary Fig. 5: Photocleavable modification allows for locally defined breakdown of DNA microbeads. a) Schematic illustration of DNA microbead design with an internal photocleavable group (PC) in the DNA linker sequence. b) Representative confocal images (λ_{ex} = 561 nm, Cy3-labeled DNA) of a PC-modified DNA microbead before (i) and after (ii) irradiation with a 405 nm laser showing that only the irradiated DNA microbead was broken down by the laser. Scale bar: 20 µm.

Supplementary Figure 6

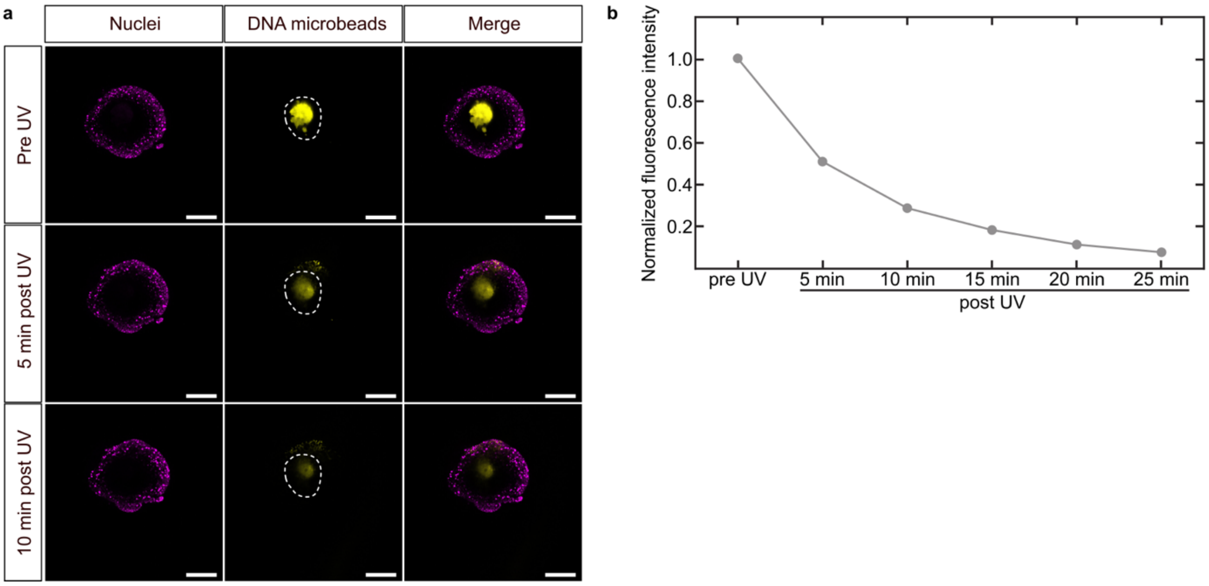

Supplementary Fig. 6: Photocleavable modification allows for the non-invasive removal of DNA microbeads from standard sized RO after their integration. a) Representative confocal images of live nuclei stained, PC-modified DNA microbeads microinjected RO before (pre UV), 5 min after (5 min post UV) and 10 min after (10 min post UV) exposure to UV light. Dashed white lines outline the DNA microbeads' positions. Scale bars: 100 µm. b) Normalized fluorescence intensity of the DNA microbeads shown in A was plotted in 5 min steps over 25 min post UV exposure.

Supplementary Figure 7

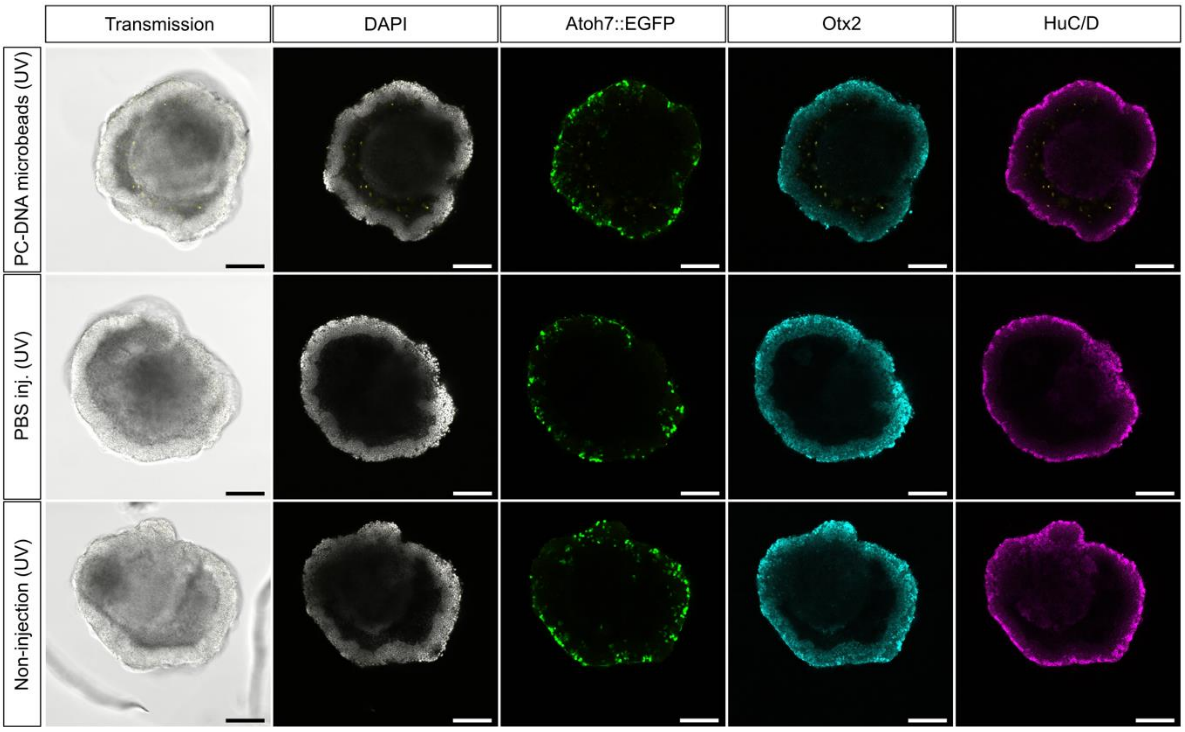

Supplementary Fig. 7: PC-modified DNA microbeads in RO development. Representative confocal images of whole-mount antibody stained day 4 RO (DAPI [nuclei], Atoh7::EGFP [retinal ganglion cells], Otx2 [bipolar cells and photoreceptors] and HuC/D [amacrine and retinal ganglion cells]) after microinjection with PC-modified DNA microbeads (PC-DNA microbeads (UV)), 1x PBS (PBS inj. (UV)) or being left uninjected (non-injection (UV)). All conditions were exposed to the same UV light regime sufficient to trigger the DNA microbead breakdown. The microinjection and DNA microbead breakdown were conducted on late day 1 at the same timepoint used for the Wnt-surrogate release experiments in Figure 4. Representative images from n = 25 organoids across 3 independent experiments. For comparison to non-UV-treated control RO see Figure 2D. Scale bars: 100 µm.

Supplementary Figure 8

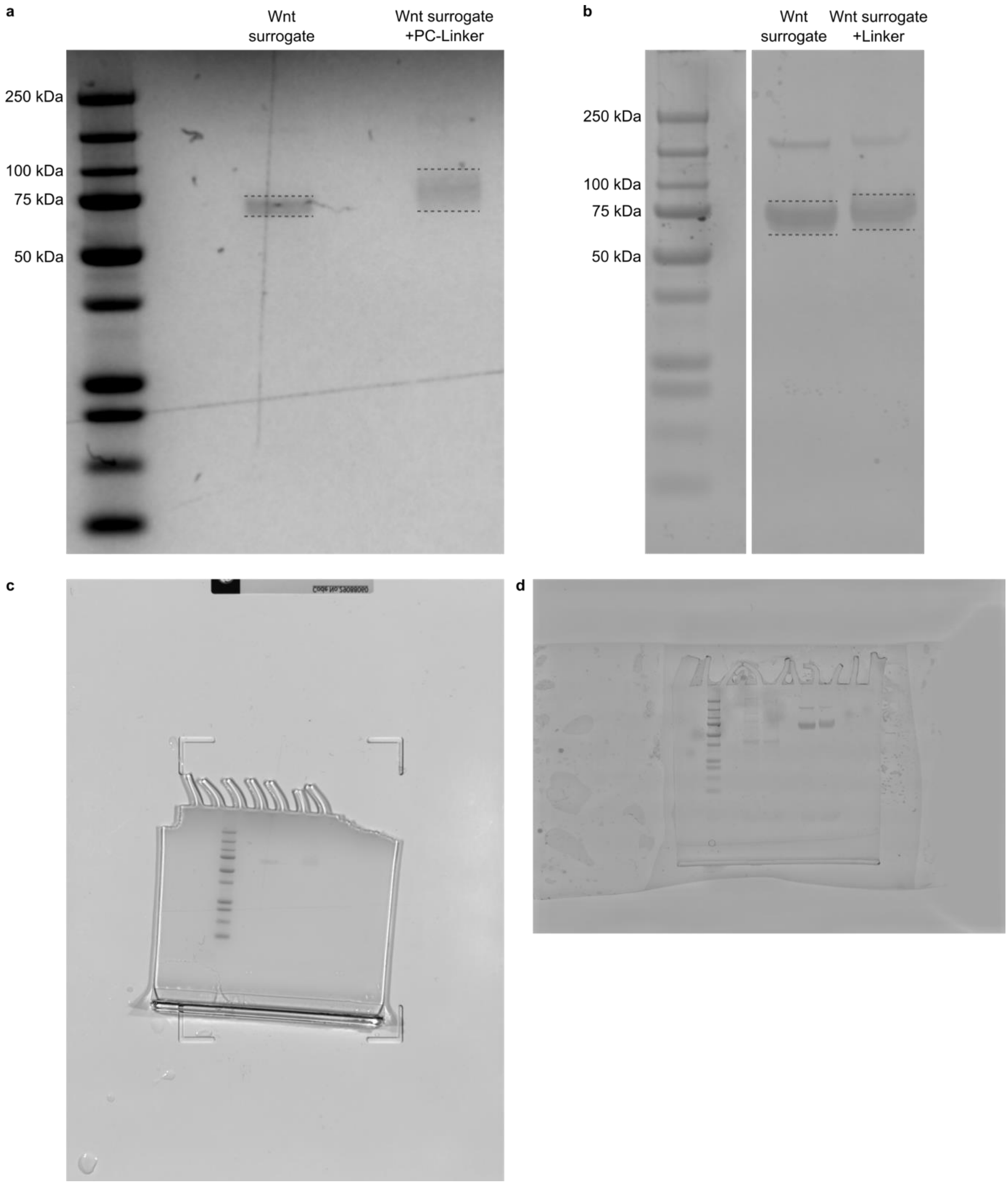

Supplementary Fig. 8: Sodium dodecyl sulfate polyacrylamide gel electrophoresis (SDS-PAGE) of the Wnt-surrogate-modified PC-linker and DNA linker. a) Image of an SDS-PAGE showing the non-reacted Wnt-surrogate and the PC-DNA linker-modified Wnt-surrogate. b) Image of an SDS-PAGE showing the non-reacted Wnt-surrogate and the DNA linker-modified Wnt-surrogate. Dashed lines indicate the lower and upper boundaries of the relevant protein bands. c) Unprocessed image of gel data presented in a). d) Unprocessed image of gel data presented in b). The relevant bands presented in b) are on the far right of the gel. The two additional

bands in the center are corresponding to a different protein, not relevant to the present study.

Supplementary Figure 9

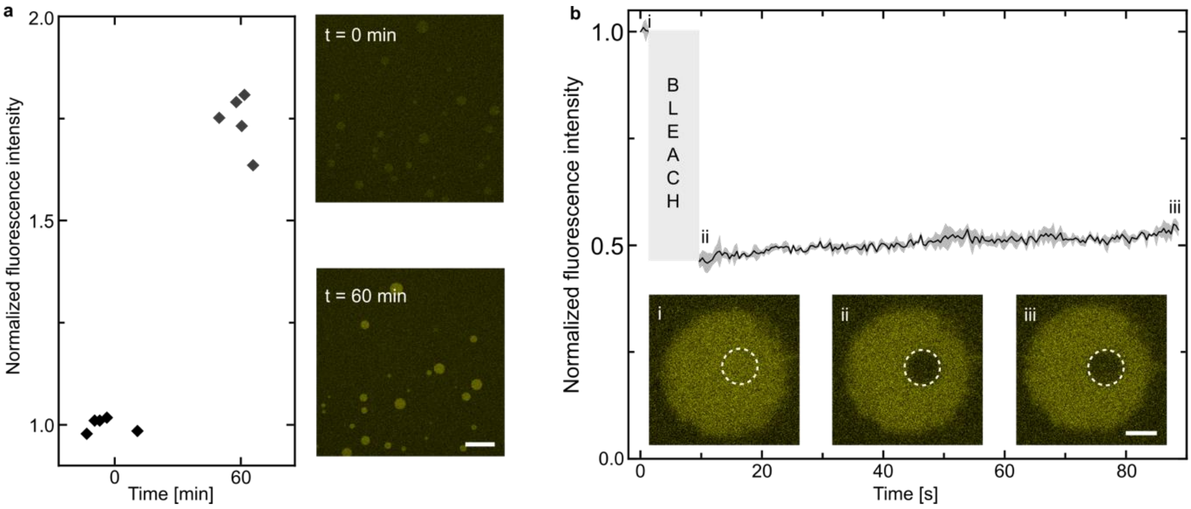

Supplementary Fig. 9: Uptake of modified DNA Y-motifs by DNA microbeads. a) Graph showing the fluorescence intensity of Cy3-modified YB-motifs upon their uptake into unlabeled DNA microbeads (individual data points shown, n = 5). Confocal microscopy images (λ_{ex} = 561 nm, Cy3-labeled Y-motif, yellow) of DNA microbeads at t = 0 min and t = 60 min of incubation of unlabeled microbeads with labeled Y-motifs. Scale bar: 50 µm. b) Graph showing the fluorescence recovery after photobleaching behavior of originally unlabeled DNA microbeads (mean ± standard deviation, n = 3) following 1 hour of incubation with Cy3-labeled DNA Y-motifs. Inserted confocal micrographs (λ_{ex} = 561 nm, Cy3-labeled Y-motif, yellow) before (i) and after bleaching (ii, iii). Scale bar: 5 µm.

Supplementary Figure 10

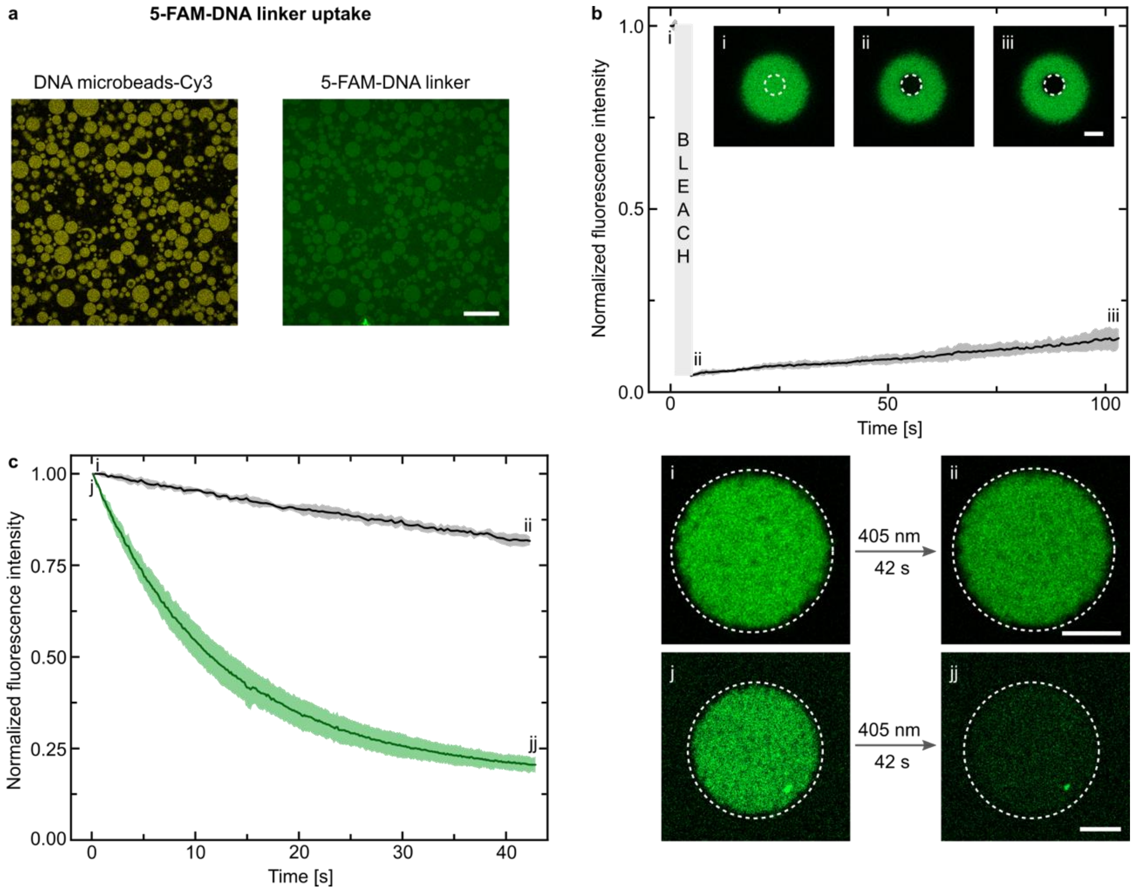

Supplementary Fig. 10: Release of 5-fluorescein-amidite (5-FAM) from intact DNA microbeads. a) Confocal microscopy images (λ_{ex} = 561 nm, Cy3-labeled Y-motif, yellow and λ_{ex} = 488 nm, 5-FAM-labeled linker, green) of DNA microbeads directly after mixing with 5-FAM-labeled linker, depicting immediate uptake of 5-FAM-labeled linker. Scale bar: 100 µm. b) Graph showing the fluorescence recovery after photobleaching behavior of the 5-FAM signal in the DNA microbeads following three washing steps with 1x PBS (mean ± standard deviation, n = 3). Inserted confocal micrographs (λ_{ex} = 488 nm, 5-FAM-labeled linker, green) before (i) and after bleaching (ii, iii). Scale bar: 10 µm. c) Graph showing the fluorescence intensity of 5-FAM inside DNA microbeads during irradiation with a 405 nm laser for non-photocleavable 5-FAM (grey line, mean ± standard deviation, n = 3) and photocleavable 5-FAM (green line, mean ± standard deviation, n = 3). Insets show confocal microscopy images (λ_{ex} = 488 nm, 5-FAM-labeled linker, green) of non-photocleavable 5-FAM modified DNA microbeads before (i) and after (ii) irradiation with a 405 nm laser, as well as confocal microscopy images (λ_{ex} = 488 nm, 5-FAM-labeled linker, green) of photocleavable 5-FAM modified DNA microbeads before (j) and after (jj) irradiation with a 405 nm laser. Scale bars: 10 µm.

Supplementary Figure 11

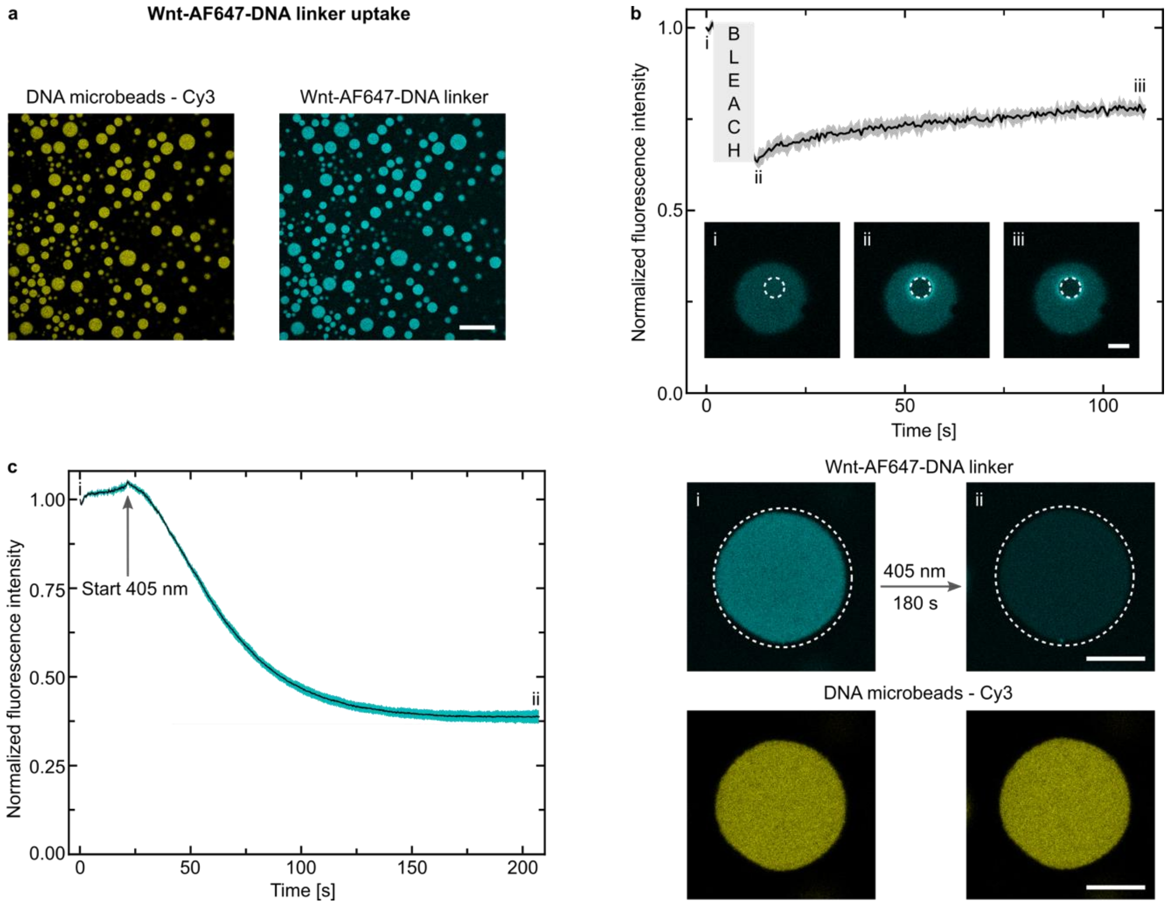

Supplementary Fig. 11: Release of Alexa-647-labeled Wnt surrogate (Wnt-AF647) from intact DNA microbeads. a) Confocal microscopy images (λ_{ex} = 561 nm, Cy3-labeled Y-motif, yellow and λ_{ex} = 640 nm, Wnt-AF647-labeled linker, cyan, respectively) of DNA microbeads after incubation with Wnt-AF647-labeled linker overnight and three washing steps with 1x PBS. Scale bar: 100 µm. b) Graph showing the fluorescence recovery after photobleaching behavior of the Wnt-AF647 signal in the DNA microbeads (mean ± standard deviation, n = 5). Inserted confocal micrographs (λ_{ex} = 640 nm, Wnt-AF647-labeled linker, cyan) before (i) and after bleaching (ii, iii). Scale bar: 10 µm. c) Graph showing the fluorescence intensity of Wnt-AF647 inside DNA microbeads during irradiation with a 405 nm laser for photocleavable Wnt-AF647-modified DNA microbeads (cyan line, mean ± standard deviation, n = 5). The onset of irradiation with a 405 nm laser is indicated. Insets show confocal microscopy images (λ_{ex} = 640 nm, Wnt-AF647-labeled linker, cyan) of photocleavable Wnt-AF647-modified DNA microbeads before (i) and after (ii) irradiation with a 405 nm laser, as well as confocal microscopy images (λ_{ex} = 561 nm, Cy3-labeled Y-motif, yellow) of the respective DNA microbead before and after irradiation. Scale bars: 20 µm.

Supplementary Figure 12

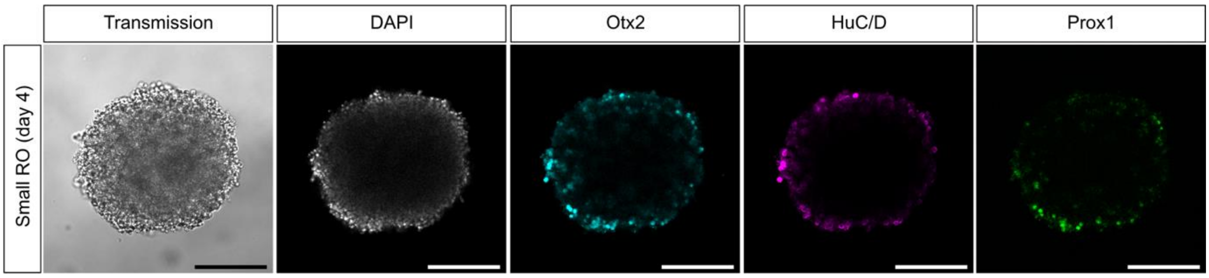

Supplementary Fig. 12: RO develop their stereotypical morphology, retinal cell type diversity and patterning even when generated as small RO. Representative confocal images of whole-mount antibody stained day 4 small RO (DAPI [nuclei], Otx2 [bipolar cells and photoreceptors], HuC/D [amacrine and retinal ganglion cells] and Prox1 [horizontal cells]). Scale bars: 100 µm.

Supplementary Figure 13

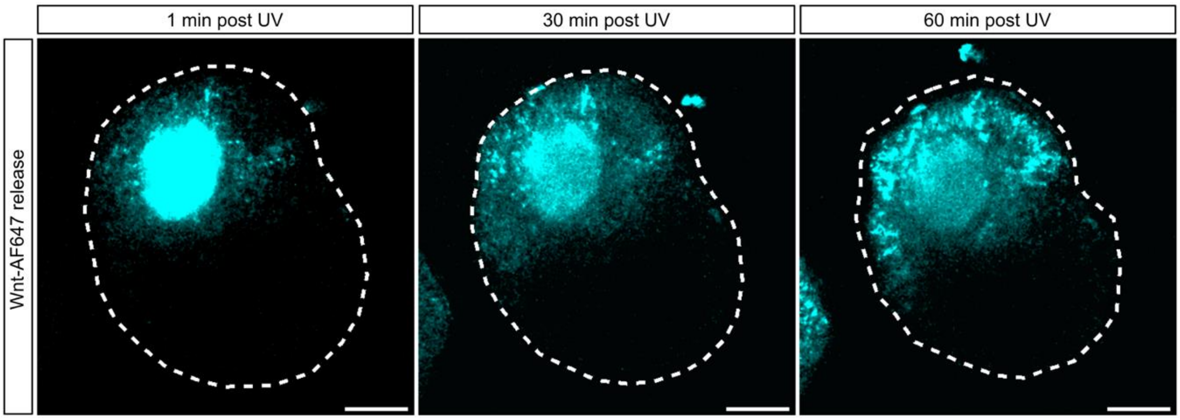

Supplementary Fig. 13: Wnt-surrogate diffusion through small RO can be restricted spatially depending on microinjection site. Representative time-lapse confocal imaging of Wnt-surrogate tagged with Alexa Fluor 647 (Wnt-AF647) after release from DNA microbeads in live small RO. Microinjection was performed deliberately closer to the RO edge (compared to Figure 3). Images show a maximum intensity z-projection of 10 slices spaced 3 µm. Z-Projections were despeckled for noise reduction (median filter 3x3 pixels). Dotted white lines indicate the small RO shape at every timepoint. Note that the shape of the small RO changes slightly between 30 min and 60 min due to minor confocal laser damage. Scale bars: 100 µm.

Supplementary Figure 14

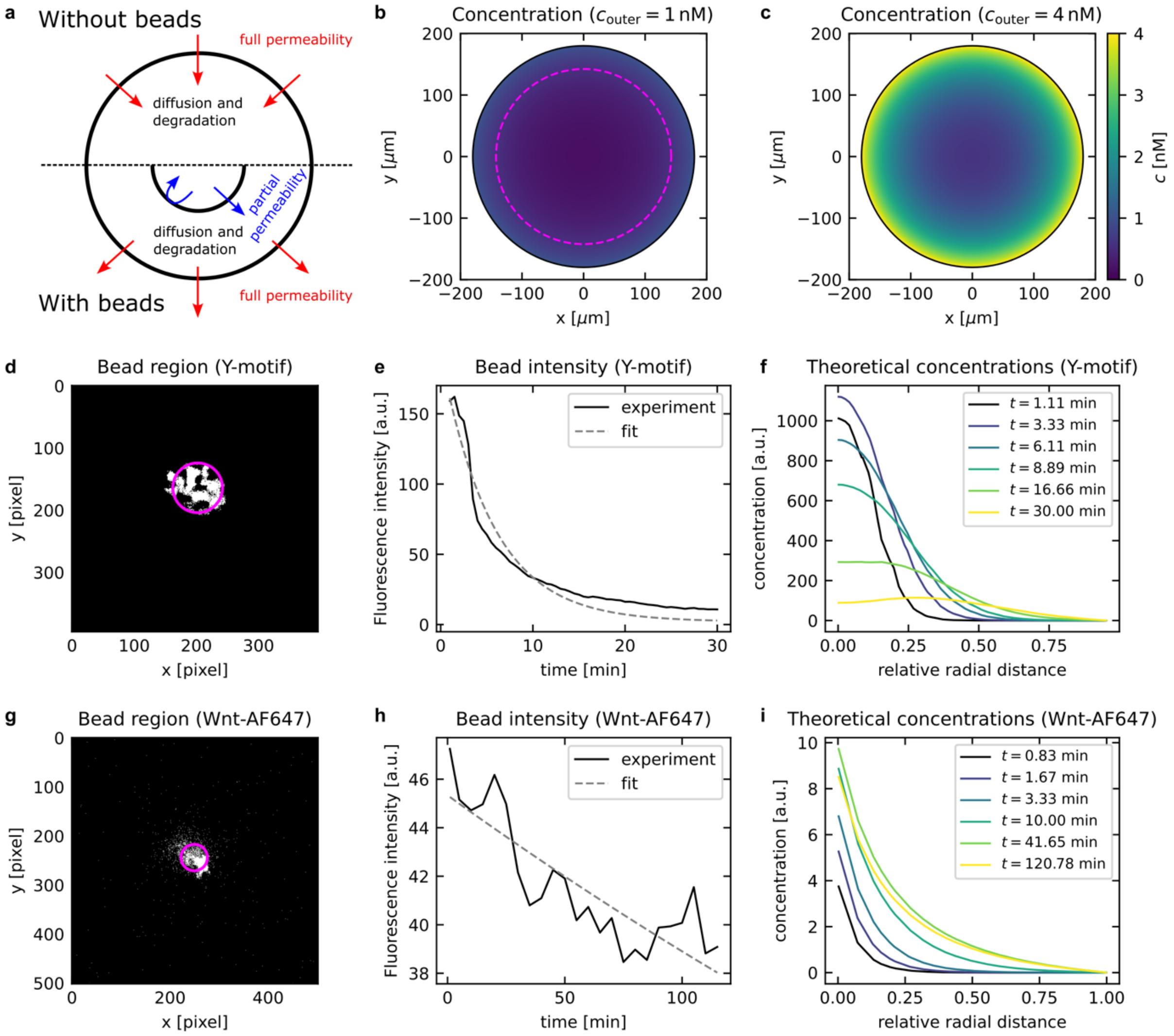

Supplementary Fig. 14: Theoretical model for the diffusion-degradation process after release. a) Schematic depiction of the three-dimensional model with and without inclusion, describing both the situation with and without beads, respectively. b, c) Simulated concentration profiles for medium-supplied Wnt for two different outer concentrations of Wnt in the medium. The magenta line in b marks the edge of the activation region with $c > 1/2\,\mathrm{nM}$. d, g) The initial images for the Y-motif and Wnt-AF647 cases with pixel intensities above the 0.98 and 0.99 intensity quantiles in white, respectively. Magenta marks the region used for intensity estimation in the inclusion around the center of mass. e, h) Average intensity in inclusion region, marked in d, g, as a function of time with exponential decay fit. f, i) Theoretical concentrations for different time points in the organoid as a function of the relative radial distance. In the Y-motif 0.0 corresponds to the organoid center and in the Wnt-AF647 0.0 corresponds to the edge of the bead. In both cases 1.0 is the organoid boundary.

Parameters: $R_0 = 100\ \mu\mathrm{m}$; f) $D_{\mathrm{Y+Wnt}} = 0.3\ \mu\mathrm{m}^2\ \mathrm{s}^{-1}$, $\kappa_{inner} = 10$, $r_0/R_0 = 0.3$, $\tilde{\mu}_{deg} = 0$; i) $D_{\mathrm{Wnt}} = 1\ \mu\mathrm{m}^2\ \mathrm{s}^{-1}$, $\kappa_{inner} = 0.02$, $r_0/R_0 = 0.3$, $\tilde{\mu}_{deg} = 5$.

Supplementary Figure 15

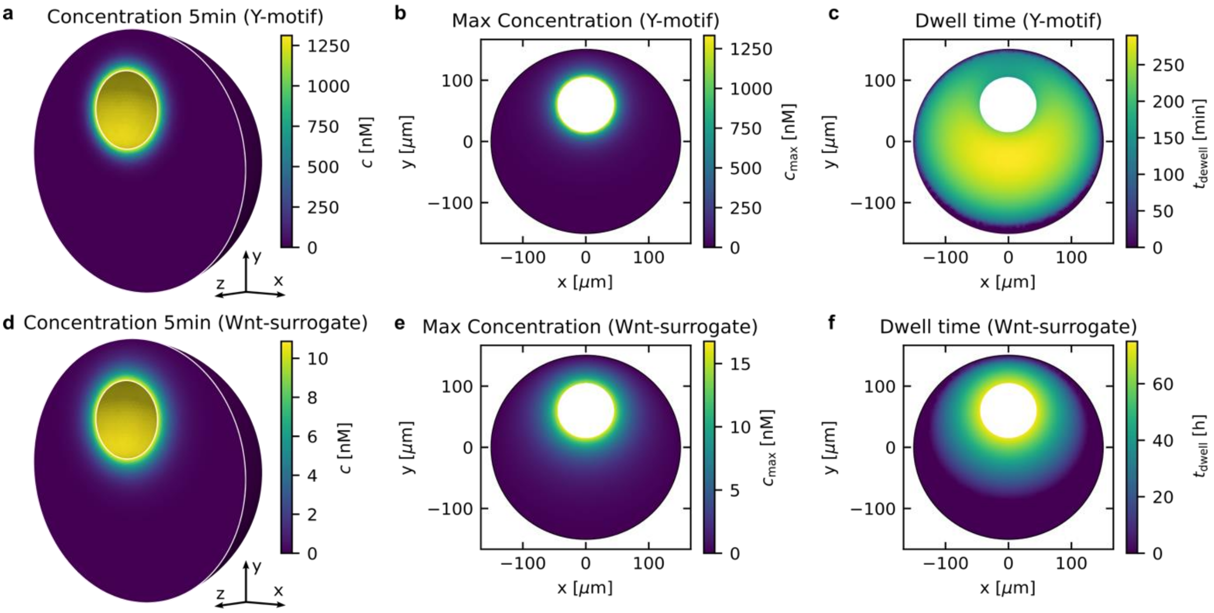

Supplementary Fig. 15: Simulated concentrations for an off-centered inclusion in organoids, as they were used in the patterning experiments, for both the Y-motif with attached Wnt-surrogate (a, b, c) and the cleaved Wnt-surrogate (d, e, f). a, d) Simulated concentrations of the morphogen 5 minutes after UV-treatment. The spherical organoid with spherical inclusion is cut through the middle plane. b, e) Maximal concentration reached after UV-treatment in the middle plane. The system is rotationally symmetric around the y-axis. c, f) Total time that morphogen is present (dwell time) and can lead to any kind of activation above the critical concentration $c_{crit} = 1/2\ \mathrm{nM}$, before it has diffused away into the surrounding medium or is degraded within the organoid. Parameters: $R_0 = 150\ \mu\mathrm{m}$; a-c) $D_{\mathrm{Y+Wnt}} = 0.3\ \mu\mathrm{m}^2\ \mathrm{s}^{-1}$, $\kappa_{inner} = 15$, $r_0/R_0 = 0.3$, $\tilde{\mu}_{deg} = 11$; d-f) $D_{\mathrm{Wnt}} = 1\ \mu\mathrm{m}^2\ \mathrm{s}^{-1}$, $\kappa_{inner} = 0.03$, $r_0/R_0 = 0.3$, $\tilde{\mu}_{deg} = 11$.

Supplementary Figure 16

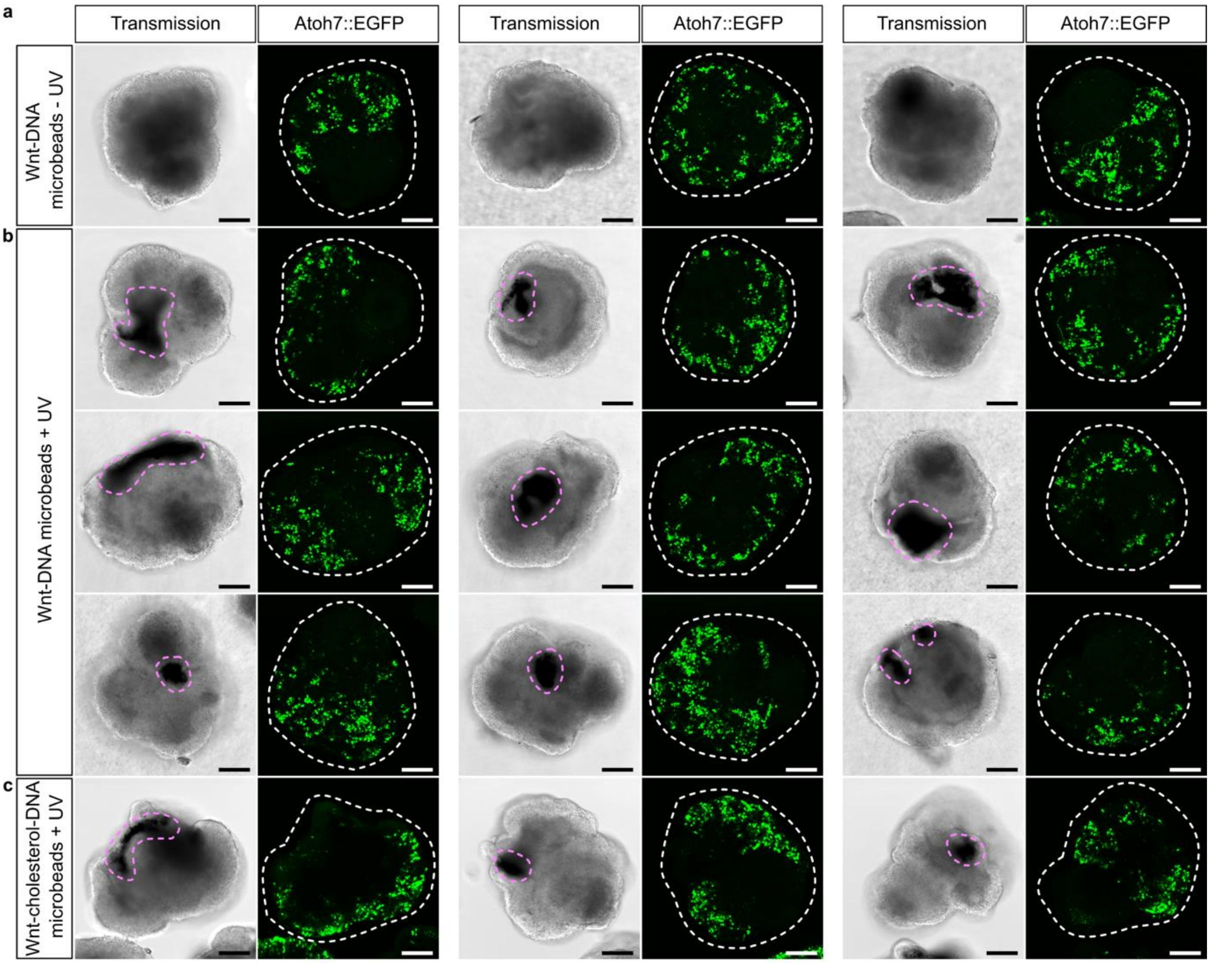

Supplementary Fig. 16: Additional representative images showing how the controlled release of Wnt-surrogate and Wnt-surrogate/cholesterol-Y-DNA-motif in RO permits the bioengineering of RO with increased and more *in vivo* like retinal cell type diversity. a, b) Representative confocal transmission images and maximum intensity z-projections (Atoh7::EGFP; 15 slices at 10 µm distance) of day 4 RO after Wnt-DNA microbead microinjection and Wnt-surrogate release at day 1 (Wnt-DNA microbeads + UV; DNA microbead design illustrated in Figure 3c). Respective controls (Wnt-DNA microbeads – UV) were left unexposed to UV and thus did not release their morphogen cargo. c) Representative confocal transmission images and maximum intensity z-projections (Atoh7::EGFP; 12 slices at 10 µm distance) images of day 4 RO after Wnt-cholesterol-DNA microbead microinjection and Wnt-surrogate/cholesterol-Y-DNA-motif release at day 1 (DNA microbead design illustrated in Figure S16). Magenta dashed lines indicate retinal pigmented epithelium, while white dashed lines outline the shape of the respective RO. Representative images taken from n = 50 organoids across 3 independent experiments. Scale bars: 100 µm.

Supplementary Figure 17

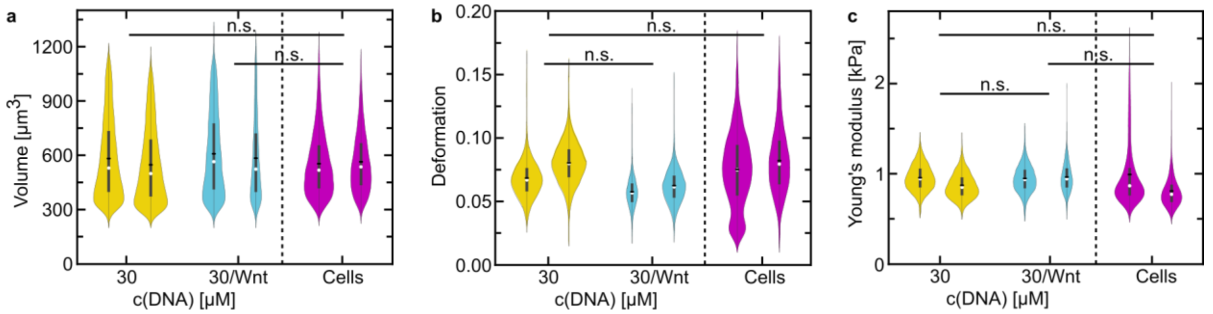

Supplementary Fig. 17: Real-time deformability cytometry of DNA microbeads with and without Wnt-surrogate and medaka retinal organoid cells. a) Volume of DNA microbeads at 30 µM DNA concentration with and without Wnt-surrogate and medaka retinal organoids. b) Deformation of DNA microbeads at 30 µM DNA concentration with and without Wnt-surrogate and medaka retinal organoids. c) Apparent Young's moduli of DNA microbeads at 30 µM DNA concentration with and without Wnt-surrogate and medaka retinal organoids. Statistical significance was assessed using a linear mixed model without adjustments (R-lme4) as integrated in Shape-Out (version 2.10.0). Statistical significance was assessed via ANOVA test. For each dataset (a-c), the data distribution is shown as a violin plot, depicting the median (white circle) and mean value (black line). The box plots depict the 25 - 75% percentile with a whisker length of 1.5 IQR. For details see SI Tables S4 - 6.

Supplementary Figure 18

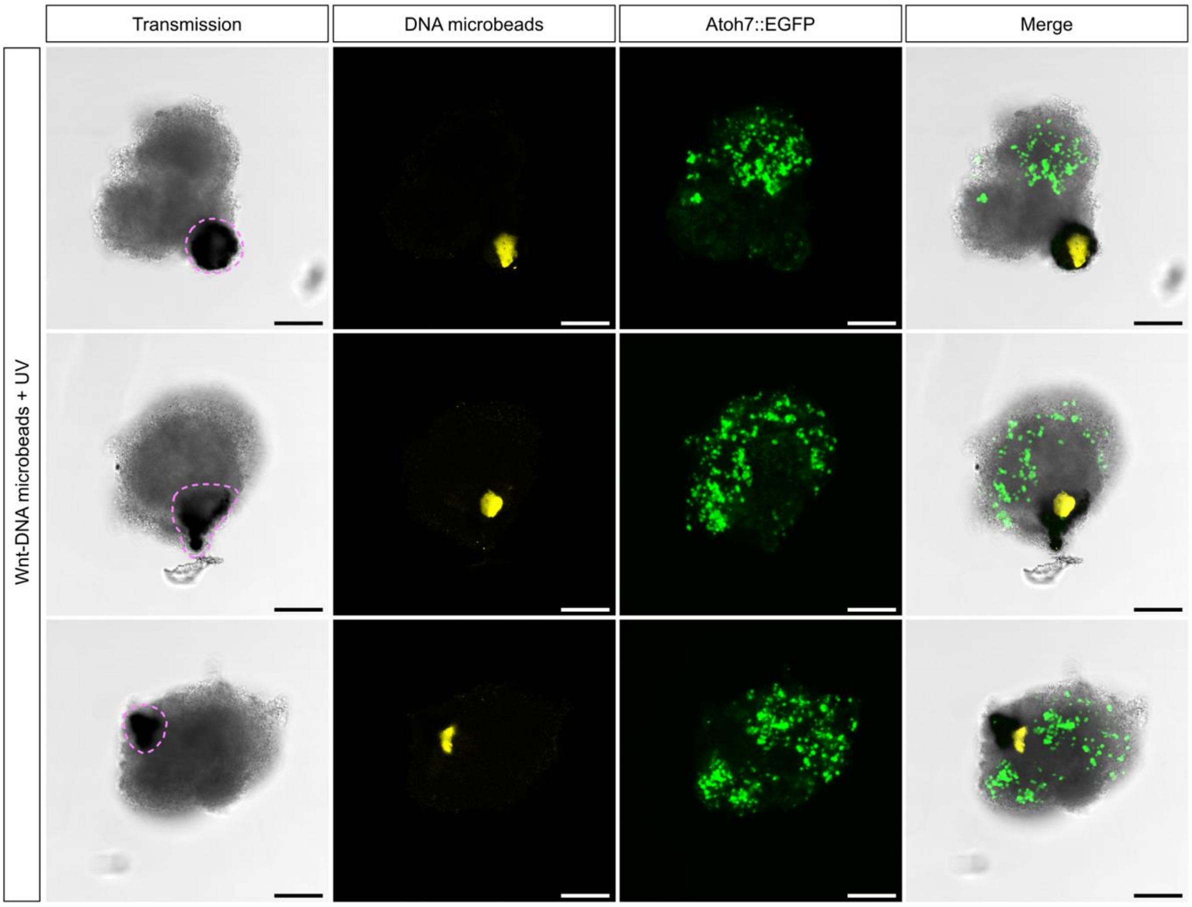

Supplementary Fig. 18: Additional representative images showing how the controlled release of Wnt-surrogate in RO permits the induction of spatially restricted RPE differentiation. Representative confocal transmission and fluorescence images and sum intensity z-projections (Atoh7::EGFP; 53 slices at 3 µm distance) of chemically fixed and stained day 4 RO after Wnt-DNA microbead microinjection and Wnt-surrogate release at day 1 (Wnt-DNA microbeads + UV; DNA microbead design illustrated in Figure 3C). Note that the RPE induction phenotype was deliberately reduced by only supplying differentiation media to the RO. Without the cell culture media change to maturation media on day 2, RO develop less RPE after induction (alongside generally being smaller). This shows the exact spatial relationship of the DNA microbead mediated Wnt-surrogate release on day 1 and the emerging RPE differentiation pattern on day 4 of the RO culture. Magenta dashed lines indicate retinal pigmented epithelium. Scale bars: 100 µm.

Supplementary Figure 19

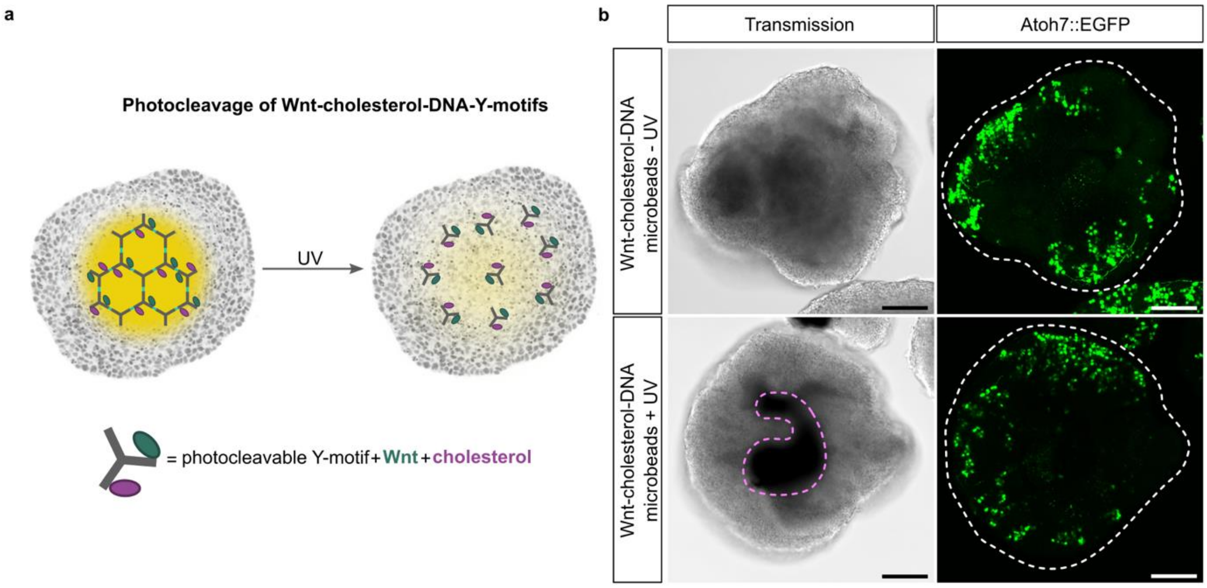

Supplementary Fig. 19: Multiple functional moieties can be released from DNA microbeads and the release can be coupled to DNA microbead breakdown. a) Schematic illustration of DNA microbead design with photocleavable Wnt-surrogate/cholesterol-Y-DNA-motif. Following UV light exposure, the DNA microbeads are broken down to release the Wnt-surrogate attached to cholesterol-modified DNA Y-motifs. b) Representative confocal transmission and maximum intensity z-projection (Atoh7::EGFP; 12 slices at 10 µm distance) images of day 4 RO after Wnt-cholesterol-DNA microbead microinjection and Wnt-surrogate/cholesterol-Y-DNA-motif release at day 1 (DNA microbead design illustrated in *a*). Magenta dashed lines indicate retinal pigmented epithelium, while white dashed lines outline the shape of the respective RO. Scale bars: 100 µm.

Supplementary Figure 20

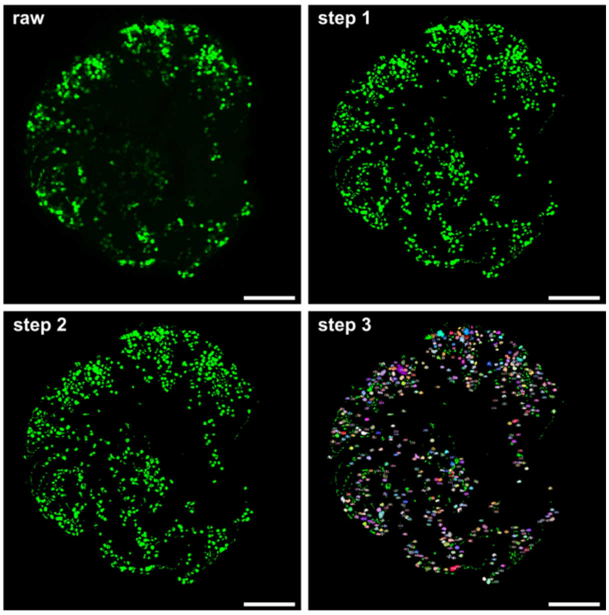

Supplementary Fig. 20: Steps described under "Quantification of area of retinal pigmented epithelium and retinal ganglion cell numbers" in the experimental section. Scale bars: 100 µm.

Supplementary Videos

Supplementary Video 1

Supplementary Video 1: Diffusion dynamics of Cy3-DNA-Y-motif after DNA microbead breakdown in live small RO. Representative time-lapse confocal imaging of Cy3-Y-motif fluorescent signal (yellow) after microinjection and subsequent UV light-mediated breakdown of PC-modified DNA microbeads in live small RO (30 s steps in between frames). Small RO are counterstained with live plasma membrane stain (magenta). Scale bar: 50 µm.

Supplementary Video 2

Supplementary Video 2: Diffusion dynamics of Wnt-AF647 after release from DNA microbeads in live small RO. Representative time-lapse confocal imaging of Wnt-surrogate tagged with Alexa Fluor 647 (Wnt-AF647) after release from DNA microbeads in live small RO (5 min steps in between frames). Images show a maximum intensity z-projection of 10 slices spaced 3 µm. Z-Projections were despeckled for noise reduction. Dotted white lines indicate the small RO shape. Scale bar: 50 µm.

Supplementary Video 3

Supplementary Video 3: Spatial correlation between DNA microbead position over time and Wnt-surrogate-release induced RPE differentiation pattern in RO. Representative automated time-lapse widefield microscopy of a DNA microbead microinjected RO between day 1 (right after Wnt-surrogate release; DNA microbead design illustrated in Figure 3c) and day 4. DNA microbeads are labeled in yellow (λ_{ex} = 561 nm, Cy3-labeled DNA), RPE is indicated by emergence of black pigment. Note that the RPE induction phenotype was deliberately reduced by only supplying differentiation media to the RO. Without the cell culture media change to maturation media on day 2, RO develop less RPE after induction (alongside generally being smaller). This shows the exact spatial correlation of the DNA microbead mediated Wnt-surrogate release on day 1 and the emerging RPE differentiation pattern on day 4 of the RO culture. Scale bar: 100 µm.

Supplementary Notes

Supplementary Note 1

Supplementary Note 1: Theoretical model of diffusing morphogens in retinal organoids

Diffusion-degradation model for externally supplied morphogens

We developed a three-dimensional diffusion-degradation model to describe both the penetration of morphogens from the medium into the organoid and from an microinjection region with the DNA microbeads into the organoid, cf. Fig. S14a. Such diffusion-degradation models have been proposed for the behavior of signaling molecules and morphogens in tissues[1, 2] and have been used to understand oxygen penetration[3]. First, we describe the diffusion of externally supplied morphogens inside the organoid. The concentration field c on the spatial domain Ω is described by

$$\partial_t c(t,x) = D\,\Delta\, c(t,x) - \mu_{deg}\; c(t,x), \qquad (1)$$

with diffusion coefficient D and degradation rate μ_{deg}, where we assumed linear degradation of morphogens by the surrounding tissue. The effective degradation rate μ_{deg} describes repeated capture and release of ligands, but with an overall tendency that ligand concentration is decreased. For simplicity the organoid is assumed to be a sphere of radius R_0. The outer boundary of the spherical organoid is $\partial\Omega_{outer}$, at which we consider radiative boundary conditions as considered in diffusion-to-capture models with partial reflection[4, 5]

$$D\nabla\, c(t,x)\,\boldsymbol{n} = -\kappa^*_{outer}\,(c(t,x) - c_{outer}) \quad \text{for } x \in \partial\Omega_{outer}. \qquad (2)$$

The diffusive flux is equal to the particle phase exchange, which will depend on the concentration differences between the different phases and the constant κ^*, which encodes the reflectiveness of the phase boundary, describing total reflection in the case of $\kappa^* = 0$ and total permeability between the two phases for $\kappa^* \to \infty$. The flux is considered with respect to the outward pointing normal $\boldsymbol{n}$.

In the case of a large morphogen reservoir in the surrounding medium we can assume c_{outer} to be constant and may solve Eq (1) for the steady state in spherical coordinates. The radial steady state solution is $c(r) = \tilde{C}\frac{\sinh(r/\rho)}{r}$, with constant $\tilde{C}$ determined from the radiative boundary condition, and it depends on the penetration depth $\rho = \sqrt{D/\mu_{deg}}$.

The solution in our case is linear in c_{outer}, which allows for a simple estimate of the penetration depth ρ from the experimental data for organoids in medium with Wnt activator. Assuming the cell patterning effect is achieved if the (steady state) concentration is above a critical concentration c_{crit}, we can estimate from the experimental images that the full organoid is (barely) activated for $c_{outer} = 4\,\text{nM}$, since we have the full differentiation pattern at this concentration. Similarly, we estimate that half the organoid is activated at $c_{outer} = 1\,\text{nM}$, since roughly half the differentiation pattern is found at this concentration in the corresponding image. This yields a spherical shell with activation above the critical concentration, cf. Fig. S14b. This implies $R_0/\rho \approx 4.11$, i.e. a penetration depth of $\rho \approx 43.8\;\mu m$ for $R_0 \approx 180\;\mu m$.

Knowing the penetration depth, we can now give an upper bound for the critical concentration in the case $\kappa^*_{outer} \to \infty$ to be $c_{crit} \approx 0.5\,\text{nM}$, matching our experimental observations. The

concentration profiles we obtain in the theoretical model in this fully permeable case are shown in Fig. S14b and c, where we have a concentration decrease toward the spherical center.

Modelling morphogen release through optical hydrogel disassembly and through Wnt-surrogate cleavage

The diffusion following the morphogen release through photocleavage (of either the DNA-Y-motif with attached Wnt-surrogate or the Wnt-surrogate itself) is described by the same diffusion-degradation model. Additionally, we now consider a spherical inclusion with radius r_0 as the region containing the microinjected morphogen carrier beads. The concentration within the inclusion, c_{inner}, is assumed to be homogeneous. Similarly to the outer boundary we consider a radiative boundary condition on the inner boundary of the inclusion, $\partial\Omega_{inner}$, cf. Eq. (2).

In the medium we now assume $c_{outer} = 0$, and the molecules are only released into the organoid from the inclusion. As the number of molecules is limited, we therefore need to explicitly consider the number of Wnt-surrogate molecules in the bead N, whose dynamics is coupled to the flux at the inner boundary, i.e.

$$\partial_t N = \kappa^*_{inner} \int_{\partial\Omega_{inner}} (c - c_{inner})\, \mathrm{d}S = \kappa^*_{inner} \int_{\partial\Omega_{inner}} c\, \mathrm{d}S - \kappa^*_{inner} \frac{N}{V_{inner}} A_{inner} \qquad (3)$$

with inclusion volume and area V_{inner} and A_{inner}, respectively.

Assuming diffusion will transport morphogen molecules away fast enough, we can neglect the latter process and approximately find an exponential decrease of the morphogen concentration in the bead region. Fitting an exponential decrease with noise offset, we find a decay time of $t_{1/e} \approx 5\ \mathrm{min}$ for the Y-motif. In the experimental data we see that a peak at finite radial distance from the center of mass (COM) forms, which propagates through the organoid, while the total intensity decreases. This is a signature of fast release and a diffusion-limited process. It corresponds to large κ^*_{inner}. Due to several processes in the release of the Y-motif, such as imperfect partial disassembly, and neglected binding dynamics and interactions of Wnt in the extracellular space, we assume that the diffusion coefficient is different than of Wnt-surrogate alone, where for Wnt diffusion coefficients of $D_{\mathrm{Wnt}} = 1 - 3\ \mu\mathrm{m}^2\ \mathrm{s}^{-1}$ have been reported[6].

We found that concentration profiles at the boundary of the central bead region of the Y-motif without Wnt-surrogate are reasonably similar to simulations with $\kappa^*_{inner} = 10$, also showing diffusion-limited behavior. Assuming that the release and diffusion behavior is similar with attached Wnt-surrogate, this corresponds to $D_{\mathrm{Y+Wnt}} = 0.3\ \mu\mathrm{m}^2\ \mathrm{s}^{-1}$ for the given decay time. Note that this theoretical diffusion coefficient considers the Y-motif with Wnt-surrogate and is therefore smaller than what we expect for the Y-motif alone.

We simulated the three-dimensional release process with the finite element method in FEniCS (details given in the corresponding section below), c.f. Fig. S14f. We find a characteristic build-up of material at finite radius.

For the Wnt-surrogate we found a decay time of $t_{1/e} \approx 600\ \mathrm{min}$ (cf. Fig. S14g, h), which is substantially larger than for the Y-motif alone. The experimental concentration profiles show an increase in profile width while retaining a sharp peak at the boundary region over time (cf. Fig. S14i). This suggests that this process is release-limited because the material can diffuse away faster than it is released. This might allow for better spatio-temporal control by the appropriate choice of the release protocol in the future.

Modelling concentrations for off-centered morphogen supply

We simulated the morphogen release from an off-centered injection region, summarized in Fig. S15. The concentrations show a gradient from the bead region into the organoid for both the cleaved DNA-Y-motif with attached Wnt-surrogate and the released Wnt-surrogate, cf. Fig. S15a, d for a concentration in the inclusion of $c_{inner} = 4000\,\mathrm{nM}$, consistent with the experiment.

In the case of the Y-motif the fast release leads to a large concentration shortly after release, reflected in the maximally reached concentration, cf. Fig. S15b. The total time where morphogen is present in the organoid, i.e. where $c > c_{crit} = 0.5\,\mathrm{nM}$, is of the order of hundreds of minutes and shows a peak in the center of the organoid, due to the diffusion-limited behavior in the corresponding spherical geometry. For the Wnt-surrogate released out of the DNA hydrogel we find smaller concentrations, which are still peaked around the inclusion. The maximally reached concentration is smaller than for the Y-motif with Wnt-surrogate, due to the slower release (cf. Fig. S15e). The release-limited nature leads to a more strongly pronounced gradient from the bead region throughout the process and therefore the total time of present morphogen, of the order of 70 hours, is also more localized around the bead (cf. Fig. S15f).

Our results suggest that indeed gradients from the bead can be created and that morphogens remain within the organoids for biologically relevant time scales.

Details of the finite element implementation of the model

We non-dimensionalized length by the organoid radius R_0, time by the diffusion time-scale R_0^2/D, and concentration by a reference concentration c_0, which we usually set through either c_{outer} or c_{inner}. The non-dimensional constants then read $\tilde{\mu}_{deg} = \frac{R_0^2}{\rho^2} = \frac{R_0^2\,\mu_{deg}}{D}$, for the dimensionless degradation rate, and $\kappa_{inner} = \frac{R_0 \kappa_{inner}^*}{D}$, for the dimensionless reflectiveness.

The inclusion concentration c_{inner} is scalar and subject to an ordinary differential equation (ODE), while the diffusing morphogen concentration is subject to a partial differential equation (PDE), cf. Eqs. (1-3). We find the weak formulation of the PDE by multiplying with test function v, integrating on the domain and using partial integration. To integrate in time we perform an implicit Euler step. To implicitly solve for c_{inner} in the time step we consider a product finite element space with additional real unknown (i.e. trial function with one degree of freedom) ω. The following equality must be fulfilled for arbitrary test function v and $r \in \mathbb{R}$, denoting the previous timestep with superscript (-1):

$$0 = \int_{\Omega} \frac{c - c^{(-1)}}{\Delta t}\, v \;\mathrm{d}V + \int_{\Omega} \nabla c \cdot \nabla v \;\mathrm{d}V + \int_{\Omega} \tilde{\mu}_{deg}\, c\, v\, \mathrm{d}V - \int_{\partial\Omega_{\mathrm{inner}}} \kappa_{inner}\, c\, v\, \mathrm{d}S$$

$$+ \int_{\partial\Omega_{\mathrm{inner}}} (c - \omega)\, r\, \mathrm{d}S$$

$$+ \int_{\partial\Omega_{\mathrm{inner}}} \kappa_{inner} \left[\frac{r_0}{r_0 + 3\kappa_{inner}\,\Delta t}\, c_{inner}^{(-1)} + \frac{r_0\,\kappa_{inner}\,\Delta t}{r_0 + 3\kappa_{inner}\,\Delta t} \frac{1}{V_{inner}}\, A_{inner}\,\omega \right] v\; \mathrm{d}S. \qquad (4)$$

The second line introduces the average of c on the inner boundary via the globally constant Lagrange multiplier ω. This Lagrange multiplier can then be used to represent the integral in the implicit ODE integration of the inclusion concentration c_{inner} in the third line.
This full weak form of the problem was implemented in FEniCS using mixed finite elements[7,8] with linear Lagrange elements (with the outer Dirichlet boundary condition) for c and a real number for ω. The three-dimensional mesh was created using Gmsh[9].

Supplementary Note References

1. Kicheva A, Pantazis P, Bollenbach T, Kalaidzidis Y, Bittig T, Jülicher F*, et al.* Kinetics of morphogen gradient formation. *Science* 2007, **315**(5811)**:** 521-525.

2. Binder P, Schnellbächer ND, Höfer T, Becker NB, Schwarz US. Optimal ligand discrimination by asymmetric dimerization and turnover of interferon receptors. *Proceedings of the National Academy of Sciences* 2021, **118**(37)**:** e2103939118.

3. Popel AS. Theory of oxygen transport to tissue. *Crit Rev Biomed Eng* 1989, **17**(3)**:** 257-321.

4. Berg HC. *Random Walks in Biology*. Princeton University Press, 1993.

5. Lindenberg K, Metzler R, Oshanin G. *Chemical Kinetics: Beyond the Textbook*, 2019.

6. Ng XW, Teh C, Korzh V, Wohland T. The Secreted Signaling Protein Wnt3 Is Associated with Membrane Domains In Vivo: A SPIM-FCS Study. *Biophys J* 2016, **111**(2)**:** 418-429.

7. M. Alnæs JB, J. Hake, A. Johansson, B. Kehlet, A. Logg, C. Richardson, J. Ring, M. E. Rognes, G. N. Wells. The FEniCS Project Version 1.5. *Archive of Numerical Software* 2015, **3**.

8. Boffi D, Brezzi F, Fortin M. *Mixed Finite Element Methods and Applications*. Springer Berlin Heidelberg, 2013.

9. Geuzaine C, Remacle J-F. Gmsh: A 3-D finite element mesh generator with built-in pre- and post-processing facilities. *International Journal for Numerical Methods in Engineering* 2009, **79**(11)**:** 1309-1331.

Supplementary Methods

Confocal fluorescence microscopy of DNA microbeads

For imaging of the DNA microbeads, an LSM 900 confocal fluorescence microscope (Carl Zeiss AG) was used. For each experiment, the pinhole size was set to one Airy unit and a Plan-Apochromat 20x/0.8 Air M27 objective was utilized. All imaging was conducted at 22°C room temperature. For imaging, the DNA microbeads were deposited into custom-built observation chambers made from glass slides (Carl Roth) attached via double-sided sticky tape (Tesa) and sealed using two-component glue (twin-sil, Picodent). Prior to assembly of the observation chamber, the glass slides were coated for 5 min with poly(vinyl-alcohol) (50 mg/mL, Sigma Aldrich).

Sodium dodecyl sulfate polyacrylamide gel electrophoresis

Sodium dodecyl sulfate polyacrylamide gel electrophoresis (SDS-PAGE) was conducted using 4 - 12 % NuPAGE gels (Thermo Fisher Scientific). Prior to SDS-PAGE, the protein samples were mixed with NuPAGE denaturing agent and loading dye (1x final concentration each) and incubated at 95°C for 5 min to denature. Per lane, 0.65 µM of protein were applied. The samples were then loaded onto the gel and run on ice for 45 min at 150 V using 10 µL Precision Plus Protein Standards All Blue (Bio-Rad) as ladder. The gels were stained under constant shaking at 80 rpm on an Orbital Shaker D0S-10L (NeoLab) using 50 mL Coomassie stain (BioRad) overnight and imaged using a c600 gel imager (Azure Biosystems).

Formation of DNA microbeads with photocleavable Wnt-modified Y-motifs

After a suspension of photocleavable DNA microbeads was passed through a 20 µm filter, 30 µL of this suspension of photocleavable DNA microbeads (60% photocleavable linker DNA microbeads) were pelleted using a C1008-GE myFUGE mini centrifuge (Benchmark Scientific) for 2 min. Then, 20 µL of supernatant were removed to leave 10 µL of a DNA microbead pellet in the reaction tube.

Separately, cholesterol-modified YA-motifs were prepared by mixing the three respective single-stranded DNA strands YA-1-chol (cholesterol-modified YA-1 strand), YA-2 and YA-3 at equimolar ratios to yield a final concentration of the resulting Y-motif of 150 µM in a 1x PBS solution. Prior to mixing, the cholesterol-modified DNA single strands were incubated at 65°C for 1 min to break up any aggregates of the cholesterol. The Y-motifs were then annealed as described above. All Y-motifs prepared this way thus harbored one cholesterol moiety.

To yield cholesterol-Wnt modified DNA microbeads, the cholesterol-tagged YA-motifs were mixed 1:3 with Wnt-surrogate-modified DNA linkers (DBCO-linker, see Supplementary Table 7) and incubated together for 10 min to allow for the linkers to bind to the Y-motifs. 10 µL of this solution were then added to the previously formed pellet of photocleavable DNA microbeads and mixed thoroughly. The final concentration of the Wnt-surrogate modified DNA linker was again 4 µM. The mixture was then incubated overnight and the microbeads likewise

washed three times using 100 µL of a 1x PBS solution to remove any excess and non-incorporated DNA Y-motifs and proteins, yielding a final volume of 10 – 15 µL of modified DNA microbeads after removal of the washing solution after centrifugation. Formation of DNA microbeads with cholesterol and Alexa Fluor 647-labeled Wnt-surrogate was conducted in the same way using Wnt-AF647-modified DNA linkers.

Formation of DNA microbeads with photocleavable 5-fluorescein-amidite (5-FAM)

5-fluorescein-amidite-azide (5-FAM) in DMSO (Lumiprobe, 10 mM stock solution) was mixed in a 1:1 molar ratio with photocleavable DBCO-modified DNA linker (80 µM) and incubated at 4°C for 72 h to yield photocleavable 5-FAM modified DNA linkers following the click reaction of the 5-fluorescein-amidite-azide to DBCO.

To create DNA microbeads with photocleavable 5-FAM modification, 30 µL of a suspension of DNA microbeads were pelleted using a C1008-GE myFUGE mini centrifuge (Benchmark Scientific) for 2 min. Then, 20 µL of supernatant were removed to leave 10 µL of DNA microbead pellet in the reaction tube. Next, the modified DNA linker with photocleavable 5-FAM was added at a final concentration of 4 µM to the DNA microbead pellet and the mixture incubated overnight to ensure full take-up of the modified linkers into the microbeads. The DNA microbeads were then washed three times using 100 µL of a 1x PBS solution to remove any excess and non-incorporated 5-FAM DNA linkers.

As a control, DBCO-modified DNA linkers without a photocleavable moiety were likewise modified with 5-FAM and added to DNA microbeads in the same fashion as above.

Quantification of the release of 5-fluorescein-amidite (5-FAM) from DNA microbeads

To quantify the release of 5-FAM from the DNA microbeads, the microbeads (n = 3) were illuminated with a 405 nm laser at 10% power (5 mW maximum power) and irradiated for 42 s until the 5-FAM signal was depleted. The frame time was set to 148.95 ms and the pixel size of the acquired image to 256 x 256 px during imaging. The mean fluorescence signal of the 5-FAM dye within the DNA microbeads was then measured using the circle tool in Fiji (NIH[1]) across all frames. All data was normalized to the mean fluorescence detected in the first frame of each video and plotted using OriginPro 2021 - Update 6 (Origin Lab Corporation). DNA microbeads harboring non-photocleavable 5-FAM modified linkers were exposed to the same conditions (n = 3 microbeads) and their fluorescence signal likewise analyzed and plotted as a control to verify whether the 5-FAM depletion is due to bleaching or release.

Sample preparation and workflow for microindentation

24 Well Glass bottom Plates (Cellvis) were treated with oxygen plasma under vacuum (0.5 mbar pressure, 200 W) for 3 min using a 300 Semi-auto plasma processor (PVA TePla AG). To coat, the wells were next filled with 1 mL of a 0.5 mg/mL poly-l-lysine (MW = 150 kDa - 300 kDa, Sigma Aldrich) solution in MilliQ water, and incubated for 60 min. The wells were then washed three times using 1x PBS solution. After a suspension of DNA microbeads was

passed through a 20 µm filter, 100 µL of DNA microbeads in 1x PBS were then added to a total volume of 1 mL 1x PBS in the wells and allowed to settle and adhere to the poly-l-lysine coated wells for 30 min before washing the wells three times using 1x PBS.

Indentation experiments were conducted on a Pavone microindenter (Optics11Life). the single microbead measurements, a cantilever with a tip size of 3.5 µm and a spring constant of 0.019 N/m was used.
For each sample, measurements on five separate DNA microbeads were undertaken. The samples were measured as triplicates. Calculation of the effective elastic moduli was based on Hertz-model fits of the indentation curve fitted to an indentation depth equal to 16% of the cantilever-tip diameter. Further, each sample was indented no further than 5% of the sample diameter, in order to exclude any measurement artifacts based on the underlying substrate.
The data of the microindentation experiments is presented in Supplementary Fig. 2 as the mean ± the standard deviation of the resulting values for the effective elastic modulus of the DNA microbeads. Data analysis was conducted using the analysis software DataViewer (V2.5.0, Optics11Life). The plot in Supplementary Fig. 2 was created using OriginPro 2021 - Update 6 (Origin Lab Corporation).

Fluorescence recovery after photobleaching

Fluorescence recovery after photobleaching (FRAP) experiments were conducted and analyzed as outlined in a previous publication[2].

Quantification of Cy3-labeled Y-motif uptake into unlabeled DNA microbeads

Unlabeled DNA microbeads were incubated with Cy3-labeled Y-motifs. Upon addition of the labeled DNA Y-motifs, the DNA microbeads were immediately imaged for 60 min. The mean fluorescence signal within DNA microbeads (n = 5) was then measured using the circle tool in Fiji (NIH[1]) at t = 0 min and t = 60 min. All data was normalized to the mean fluorescence detected at t = 0 min and plotted using OriginPro 2021 - Update 6 (Origin Lab Corporation). After imaging, FRAP measurements were conducted on three independent DNA microbeads and analyzed as described above.

Fluorescent labeling and imaging of medaka retinal organoids and embryos

For plasma membrane staining, organoids were incubated in CellMask™ Deep Red plasma membrane stain (Thermo Fisher Scientific, Cat#: C10046; 1:1000) solved in differentiation media for 30 min at room temperature (RT) and subsequently washed. Samples were additionally fixed with 4% PFA in Fig. 2b. For live nuclei staining, organoids were incubated in DRAQ5™ (Thermo Fisher Scientific, Cat#: 62251; 1:500) solved in differentiation media for 30 min at RT and subsequently washed.

For whole-mount antibody staining, organoids were fixed in 4% PFA for 3 h at RT and washed with PTW (0.05% Tween20 solved in 1x PBS). Fixed samples were permeabilized with acetone for 15 min at -20°C, blocked with 4% sheep serum, 1% BSA and 1% DMSO in PTW for 2 h at RT. Samples were incubated with primary antibodies for 48 h at 4°C. The following primary antibodies were used: chicken anti-GFP (Thermo Fisher Scientific, Cat#: A10262; 1:300), mouse anti-HuC/D (Thermo Fisher Scientific, Cat#: A21271; 1:150), goat anti-Otx2 (R&D systems, Cat#: AF1979; 1:150) and rabbit anti-Prox1 (Sigma Aldrich, Cat#: AB5475; 1:200). Samples were washed five times with PTW and subsequently incubated with the respective secondary antibodies (1:500) for 24 h at 4°C. The following secondary antibodies were used: donkey anti-chicken Alexa Fluor 488 (Jackson ImmunoResearch Europe Ltd., Cat#: 703-545-155), donkey anti-mouse Alexa Fluor 647 (Jackson ImmunoResearch Europe Ltd., Cat#: 715-605-151), donkey anti-goat Alexa Fluor 594 (Thermo Fisher Scientific, Cat#: A-11058), donkey anti-rabbit (Thermo Fisher Scientific, Cat#:A32790) and DAPI (10 µg/ml in DMSO, Carl Roth, Cat#: 6335.1). Samples were washed five times in PTW in preparation for imaging.

Epifluorescence microscopy of live organoids was conducted on a Leica DMi8 microscope. Automated time-lapse widefield microscopy (Supplementary Video 3) was conducted on the ACQUIFER Imaging Machine (ACQUIFER Imaging GmbH, Heidelberg, Germany).[3] Organoids were loaded into 96-well plates and placed at 26°C in the plate holder of the ACQUIFER machine. Brightfield and 528 nm fluorescence channel was acquired with a 20x NA 0.45 objective. Imaging was performed over 58 h of organoid development with 2 h intervals. Live (Fig. 3) or fixed organoids were either imaged with Leica TCS Sp8 confocal microscope (20x oil immersion objective) or on the multiview selective-plane illumination MuVi SPIM Multiview light-sheet microscope (Luxendo Light-sheet, Bruker Corporation).[4] Live fluorescent labeling imaging of embryos was performed on the Leica TCS Sp8 confocal microscope (20x oil immersion objective). Embryos were mounted heads down in 1% low melting point agarose (Carl Roth, Cat#: 6351.5) into a MatTek dish (Mattek, Cat#: P35G-1.5-10-C) covered with ERM + 100 U/ml penicillin-streptomycin. The gross morphology of embryos was assessed by stereomicroscopy (Nikon SMZ18).

As differentiated retinal cell types in retinal organoids are most frequently located towards the organoids rim and at the same time laser intensity is lost quickly during confocal microscopy illumination with depth of bigger tissues like organoids, the organoid's inside appears dimmer or even black (depending on a given fluorophore's brightness; seen in Fig. 2d, Supplementary Figs. 6, 12).

For light sheet microscopy, organoids were mounted in 1% low melting point agarose inside a FEP tube (Karl Schupp AG) fixed on glass capillaries. Four volumes (two cameras and two rotation angles) were acquired with the 16× detection setup in 2 channels: Cy3 (DNA microbeads); Far Red (CellMask Deep Red). Images were subjected to deconvolution using a theoretical point spread function.

In Fig. 2d, DNA microbead fluorescent signal (Cy3) was image subtracted from Otx2 fluorescent signal (Alexa Fluor 594) prior to image overlay due to overlapping emission spectra of the spatially distinct sources of emission.

Quantification of area of retinal pigmented epithelium and retinal ganglion cell numbers

For quantification of the area of retinal pigmented epithelium in transmission confocal images, the total pixel count of all pixels with values between 0 and 10 was acquired.

For quantification of retinal ganglion cell numbers, maximum intensity z-projections of Atoh7::EGFP positive cells were first subjected to noise reduction by Despeckle (median filter 3x3 pixels). Secondly foreground objects were segmented from background by Auto Local Thresholding using Bernsen's thresholding with a radius of 5 and the default contrast threshold of 15 (Supplementary Fig. 20 step 1). Bernsen's local thresholding was selected due to different fluorescence intensities of cells dependent on reporter expression levels as well as non-uniform illumination dependent on cell positions within the 3D volume of the respective organoid. Thresholded binary images were Watershed to separate partially merged cell bodies by initial maximum intensity z-projections (Supplementary Fig. 20 step 2). Predominantly round cell bodies were automatically counted by Analyze Particle ImageJ implementation with a size specification of 15-infinity and a circularity specification of 0.4-1 (Supplementary Fig. 20 step 3). For the data in Fig. 4b an additional initial global background subtraction of 30 pixels was performed.

Two-tailed Student's *t*-test with unequal variance was used for the calculation of significant differences in Fig. 4. P-values < 0.05 were considered statistically significant. Data distribution was assumed to be normal but this was not formally tested.

Supplementary Methods References

1. Schneider CA, Rasband WS, Eliceiri KW. NIH Image to ImageJ: 25 years of image analysis. *Nature Methods* 2012, **9**(7)**:** 671-675.

2. Walther T, Jahnke K, Abele T, Göpfrich K. Printing and Erasing of DNA-Based Photoresists Inside Synthetic Cells. *Advanced Functional Materials* 2022, **32**(25)**:** 2200762.

3. Pandey G, Westhoff JH, Schaefer F, Gehrig J. A Smart Imaging Workflow for Organ-Specific Screening in a Cystic Kidney Zebrafish Disease Model. *International Journal of Molecular Sciences* 2019, **20**(6)**:** 1290.

4. Krzic U, Gunther S, Saunders TE, Streichan SJ, Hufnagel L. Multiview light-sheet microscope for rapid in toto imaging. *Nat Methods* 2012, **9**(7)**:** 730-733.
