# Supplementary material for: DNA microbeads for spatio-temporally controlled morphogen release within organoids
Source: Nat Nanotechnol. 2024 Sep 9;19(12):1849–57. doi: 10.1038/s41565-024-01779-y (PMC11638066; doi:10.1038/s41565-024-01779-y)
Supplement: Supplementary file 2 — Reporting Summary [file 41565_2024_1779_MOESM2_ESM.pdf]

nature portfolio

Corresponding author(s): Joachim Wittbrodt, Kerstin Göpfrich

Last updated by author(s): Aug 1, 2024

Reporting Summary

Nature Portfolio wishes to improve the reproducibility of the work that we publish. This form provides structure for consistency and transparency in reporting. For further information on Nature Portfolio policies, see our Editorial Policies and the Editorial Policy Checklist.

Statistics

For all statistical analyses, confirm that the following items are present in the figure legend, table legend, main text, or Methods section.

n/a	Confirmed	
☐	☒	The exact sample size (*n*) for each experimental group/condition, given as a discrete number and unit of measurement
☐	☒	A statement on whether measurements were taken from distinct samples or whether the same sample was measured repeatedly
☐	☒	The statistical test(s) used AND whether they are one- or two-sided<br>*Only common tests should be described solely by name; describe more complex techniques in the Methods section.*
☒	☐	A description of all covariates tested
☒	☐	A description of any assumptions or corrections, such as tests of normality and adjustment for multiple comparisons
☐	☒	A full description of the statistical parameters including central tendency (e.g. means) or other basic estimates (e.g. regression coefficient) AND variation (e.g. standard deviation) or associated estimates of uncertainty (e.g. confidence intervals)
☐	☒	For null hypothesis testing, the test statistic (e.g. *F*, *t*, *r*) with confidence intervals, effect sizes, degrees of freedom and *P* value noted<br>*Give P values as exact values whenever suitable.*
☒	☐	For Bayesian analysis, information on the choice of priors and Markov chain Monte Carlo settings
☒	☐	For hierarchical and complex designs, identification of the appropriate level for tests and full reporting of outcomes
☒	☐	Estimates of effect sizes (e.g. Cohen's *d*, Pearson's *r*), indicating how they were calculated

Our web collection on statistics for biologists contains articles on many of the points above.

Software and code

Policy information about availability of computer code

Data collection	cSeries Capture Software (Azure Biosystems c600), ZEN blue Versoin 3.1 (Carl Zeiss AG), ShapeIn (version 2.2.2.4, Zellmechanik Dresden), PavoneV1.10.1 (Optics11Life); Confocal microscopy (Leica LAS X, Version 3.5.7.23225); Epifluorescence microscopy (Leica LAS X, Version 3.3.3.16958); Stereomicroscopy (Nikon, DS-Ri1-U3 (Version 1200.0005.00.20.0201.0208.01000000)); Automated widefield microscopy (Acquifer, Version 6.4.0.18671), FEniCS (2019.1)
Data analysis	ShapeOut (version 2.10.0, Zellmechanik Dresden), ImageJ (versions 1.54f & 2.14.0/1.54f), Inkscape (versions 1.0.1 & 1.2.2), Origin Pro 2021 - Update 6 (Origin Lab Corporation), DataViewer V2.5.0 (Optics11Life), Microsoft Excel (Version 16.65), Python (Version 3.10 & 3.12), Tifffile (2024.7.2), Scipy (1.13), Pandas (2.2), Fiji (1.53f51), MultiStackReg (1.5), Matplotlib (Version 3.7.0), Seaborn (Version 0.11.2)

For manuscripts utilizing custom algorithms or software that are central to the research but not yet described in published literature, software must be made available to editors and reviewers. We strongly encourage code deposition in a community repository (e.g. GitHub). See the Nature Portfolio guidelines for submitting code & software for further information.

Data

Policy information about availability of data

All manuscripts must include a data availability statement. This statement should provide the following information, where applicable:

- Accession codes, unique identifiers, or web links for publicly available datasets
- A description of any restrictions on data availability
- For clinical datasets or third party data, please ensure that the statement adheres to our policy

The datasets generated during and analysed during the current study are available in the [Research Data] repository on heiDATA /Göpfrich Group - Biophysical Engineering of Life with the identifier https://doi.org/10.11588/data/ADYUNN.

Research involving human participants, their data, or biological material

Policy information about studies with human participants or human data. See also policy information about sex, gender (identity/presentation), and sexual orientation and race, ethnicity and racism.

Reporting on sex and gender	n.a.
Reporting on race, ethnicity, or other socially relevant groupings	n.a.
Population characteristics	n.a.
Recruitment	n.a.
Ethics oversight	n.a.

Note that full information on the approval of the study protocol must also be provided in the manuscript.

Field-specific reporting

Please select the one below that is the best fit for your research. If you are not sure, read the appropriate sections before making your selection.

☒ Life sciences ☐ Behavioural & social sciences ☐ Ecological, evolutionary & environmental sciences

For a reference copy of the document with all sections, see nature.com/documents/nr-reporting-summary-flat.pdf

Life sciences study design

All studies must disclose on these points even when the disclosure is negative.

Sample size	No sample-size calculations were performed. Sample size was determined to be adequate based on the magnitude and consistency of measurable differences between groups.
Data exclusions	No data was excluded from the analysis.
Replication	All experiments were replicated with sufficient technical and experimental replicates determined by the investigators experience with the statistical analysis of common endpoints of our model system as well as the study designs of comparable studies from the literature.
Randomization	Organoids were distributed randomly to all conditions.
Blinding	Investigators were not blinded as it was not compatible with the experimental procedure.

Reporting for specific materials, systems and methods

We require information from authors about some types of materials, experimental systems and methods used in many studies. Here, indicate whether each material, system or method listed is relevant to your study. If you are not sure if a list item applies to your research, read the appropriate section before selecting a response.

Materials & experimental systems

n/a	Involved in the study
	☒ Antibodies
☒	Eukaryotic cell lines
☒	Palaeontology and archaeology
☒	Animals and other organisms
☒	Clinical data
☒	Dual use research of concern
☒	Plants

Methods

n/a	Involved in the study
☒	ChIP-seq
☒	Flow cytometry
☒	MRI-based neuroimaging

Antibodies

Antibodies used

Primary antibodies:
chicken anti-GFP (Thermo Fisher Scientific, Cat#: A10262; Lot: 2480084)
mouse anti-HuC/D (Thermo Fisher Scientific, Cat#: A21271; Lot: 2441512)
goat anti-Otx2 (R&D systems, Cat#: AF1979; Lot: KNO1022091)
rabbit anti-Prox1 (Sigma Aldrich, Cat#: AB5475; Lot: 3811358)
Secondary antibodies:
donkey anti-chicken Alexa Fluor 488 (Jackson ImmunoResearch Europe Ltd., Cat#: 703-545-155; Lot: 162189)
donkey anti-mouse Alexa Fluor 647 (Jackson ImmunoResearch Europe Ltd., Cat#: 715-605-151; Lot: 105869)
donkey anti-goat Alexa Fluor 594 (Thermo Fisher Scientific, Cat#: A-11058; Lot: 714270)
donkey anti-rabbit Alexa Fluor 488 (Thermo Fisher Scientific, Cat#: A32790; Lot: VC296619)

Validation

All primary antibodies have previously been validated for both the species and the exact application shown in this study (retinal organoids derived from Oryzias latipes originated embryonic pluripotent cells) [1].

[1] Zilova L, Weinhardt V, Tavhelidse T, Schlagheck C, Thumberger T, Wittbrodt J. Fish primary embryonic pluripotent cells assemble into retinal tissue mirroring in vivo early eye development. Elife2021, 10.

Plants

Seed stocks

n.a.

Novel plant genotypes

n.a.

Authentication

n.a.
